# Hydrogen Sulfide Promotes TAM‐M1 Polarization through Activating IRE‐1α Pathway via GRP78 S‐Sulfhydrylation to against Breast Cancer

**DOI:** 10.1002/advs.202413607

**Published:** 2025-01-04

**Authors:** Mingyi Ju, Weiwei Tong, Jia Bi, Xianxin Zeng, Aoshuang Qi, Mingli Sun, Jian Wen, Lin Zhao, Minjie Wei

**Affiliations:** ^1^ Department of Pharmacology School of Pharmacy China Medical University Shenyang 110122 China; ^2^ Liaoning Key Laboratory of molecular targeted anti‐tumor drug development and evaluation China Medical University Shenyang 110122 China; ^3^ Department of Laboratory Medicine Shengjing Hospital of China Medical University Shenyang 110122 China; ^4^ Department of Breast Surgery The Fourth Affiliated Hospital of China Medical University Shenyang 110122 China; ^5^ School of Pharmacy Qiqihar Medical University Qiqihar 161006 China

**Keywords:** breast cancer, hydrogen sulfide, macrophage repolarization, S‐sulfhydrylation, tumor‐associated macrophages

## Abstract

Hydrogen sulfide (H_2_S)‐mediated protein S‐sulfhydration has been shown to play critical roles in several diseases. Tumor‐associated macrophages (TAMs) are the predominant population of immune cells present within solid tumor tissues, and they function to restrict antitumor immunity. However, no previous study has investigated the role of protein S‐sulfhydration in TAM reprogramming in breast cancer (BC). Therefore, the aim is to investigate whether protein S‐sulfhydration can regulate TAM reprogramming and its underlying mechanism in BC. The results showed that in BC, the CTH‐H_2_S axis is positively correlated with the presence of an anti‐tumor phenotype in TAMs. NaHS, as an H_2_S donor, repolarized TAMs into M1 macrophages to block the tumor‐promoting activities of TAMs both in vitro and in vivo. Mechanistically, H_2_S‐mediated S‐sulfhydration of the protein chaperone glucose‐regulated‐protein 78 (GRP78) induced endoplasmic reticulum transmembrane protein kinase‐1α (IRE‐1α) dissociation from GRP78, which enhanced the phosphatase activity of IRE‐1α itself in BC‐TAMs, while the Cys420 site mutation of GRP78 interfered with these effects. Collectively, GRP78 S‐sulfhydrylation mediated by H_2_S at the Cys420 residue decreased the tumor burden and inhibited lung metastasis of BC through reprograming TAMs via activating the IRE‐1α pathway, indicating that targeting GRP78 S‐sulfhydration represents a promising intervention for TAM‐M1 repolarization in BC.

## Graphical abstract

This work indicates that GRP78 S‐sulfhydrylation mediated by H_2_S at Cys420 residue decreases tumor burden and inhibits lung metastasis of BC through reprogramming TAMs via activating IRE‐1α pathway, which offers new leads for interventions in TAMs reprogramming in BC.

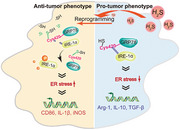

## 1 Introduction

Protein S‐sulfhydration, a recently discovered protein post‐translational modification (PTM), is increasingly recognized as a major form of protein functional modification, which is conceivably as significant as phosphorylation.^[^ ^1^ ^]^ S‐sulfhydration proteins have been reported to constitute 25%–50% of the protein content in the liver of mice.^[^ ^2^ ^]^ During sulfhydration, the reactive cysteine thiol group is modified to a hydropersulfide moiety (‐SSH). This modification increases the reactivity of cysteine residues, which is typically mediated by hydrogen sulfide (H_2_S).^[^ ^3^ ^]^ S‐sulfhydration frequently leads to a change in conformation that modifies the enzymatic activity or functional role of the protein, thereby serving as a significant switch or regulator.^[^ ^2^ ^]^ S‐sulfhydration plays a crucial role in coordinating a range of physiological processes, such as inflammation, endoplasmic reticulum stress (ERS), and signal transduction.^[^ ^4^ ^]^

H_2_S, as a gasotransmitter, can easily pass through membranes, which sets it apart from traditional signal transduction regulators, allowing it to operate autonomously without the need for transmembrane receptors.^[^ ^5^ ^]^ In mammals, H_2_S is tightly and precisely regulated by three enzymes, cystathionine β‐synthase (CBS), cystathionine γ‐lyase (‐CTH), and 3‐mercaptopyruvate sulfur transferase (3‐MST).^[^ ^6^ ^]^ H_2_S, known as the third gasotransmitter, has been recognized as a “cloud” that “surrounds” immune cells, and due to the synthesis of H_2_S from internal sources and its introduction from neighboring parenchymal cells, there is a significant impact on the viability and functionality of these cells.^[^ ^7^, ^8^, ^9^, ^10^ ^]^ Previous research has indicated that H_2_S has the potential as a therapeutic agent for mitigating proinflammatory macrophage characteristics via the S‐sulfhydration of crucial macrophage proteins in certain inflammatory conditions.^[^ ^11^, ^12^ ^]^ Previous research has reported that the S‐sulfhydration of PTP1B at Cys215 increases the phosphorylation of tyrosine 619 in PRKR‐like ER kinase (PERK) and stimulates its catalytic activation in response to ERS.^[^ ^13^ ^]^ Meanwhile, evidence shows that the ERS response regulates the STAT1 and STAT6 pathways of macrophages to facilitate their polarization toward the M1 phenotype, which exacerbates ischemia‐reperfusion injury in fatty liver.^[^ ^14^ ^]^ Moreover, inhibition of ERS has been shown to induce transformation of phenotypic M1 macrophages to M2 macrophages.^[^ ^15^ ^]^ These findings suggest that there is some cross‐talk between protein S‐sulfhydration, ERS, and macrophage reprogramming. However, the role of protein S‐sulfhydration in tumor‐associated macrophage (TAM) reprograming in the tumor microenvironment (TME) remains unclear, as does the precise mechanism of S‐sulfhydration in response to ERS.

GRP78 is a master chaperone protein that regulates the unfolded protein response (UPR) in ERS. In mammalian cells, three transmembrane proteins located in the endoplasmic reticulum (ER) function as detectors of ER activity: activating transcription factor 6 (ATF6), inositol‐requiring enzyme 1α (IRE1α), and PERK. Under proteostasis conditions, the molecular chaperone GRP78 interacts with immunoglobulin proteins to bind to specific sensors, thereby maintaining their quiescent state. During ERS, GRP78 dissociates from the sensors because of its increased affinity to misfolded or unfolded proteins, which induces their activation and subsequent activation of ERS.^[^ ^16^ ^]^ Typically, the chaperone activity of GRP78 is regulated by PTMs.^[^ ^17^, ^18^, ^19^ ^]^ Moreover, a previous study reported the presence of cysteine residues in GRP78 that can be post‐translationally oxidated to alter its chaperone activity and then activate ERS.^[^ ^20^ ^]^ However, whether GRP78 can be S‐sulfhydrated and whether the S‐sulfhydration of GRP78 can reprogram TAMs require further clarification.

Therefore, in this study, we investigated whether S‐sulfhydration of proteins involved in the ERS response process can regulate TAM reprograming in breast cancer (BC) and delved into revealing its underlying mechanism, intending to better understand whether targeting protein S‐sulfhydration can be considered a new therapeutic intervention for BC.

## 2 Results

### 2.1 CTH/H_2_S Pathway Significantly Correlates with Prognosis and TAM Polarization in BC

**Figure 1:**
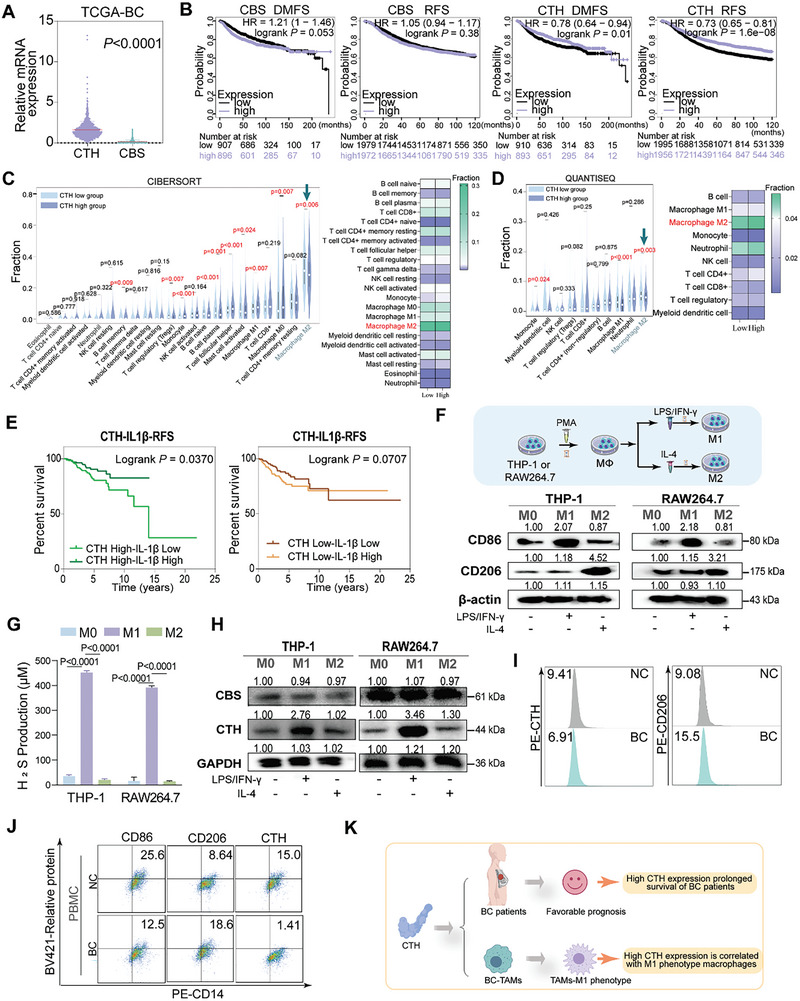
CTH as an H_2_S synthesizing enzyme was correlated with prognosis and macrophage polarization in BC. A) Comparison of CTH expression with CBS expression in BC samples obtained from TCGA‐BC database. B) Kaplan–Meier survival curves of RFS and DMFS of BC patients grouped by CBS and CTH expression Enrichment of immune cells in patients grouped by CTH expression using CIBERSORT algorithm (C) and QUANTISEQ algorithm (D) based on TCGA‐BC database. E) Kaplan–Meier survival curves of overall survival of BC patients grouped by CTH and IL‐1β expression. F) Flowchart for constructing M0, M1, and M2 macrophages in vitro using THP‐1 and RAW264.7 cells (above). M1 and M2 macrophages were successfully induced in vitro, confirmed by western blot (below). n = 3 cell samples. G) H_2_S production in M0, M1, and M2 phenotypes macrophages derived from THP‐1 and RAW264.7 cells. n = 3 cell samples. H) Protein expression of CTH and CBS in M0, M1, and M2 phenotypes macrophages derived from THP‐1 and RAW264.7 cells were detected by western blot. n = 3 cell samples. I) Flow cytometry analysis of CTH and CD206 expression in macrophages cultured with serum derived from BC patients and healthy donors. n = 3 cell samples. J) Flow cytometry analysis of CTH, CD86, and CD206 expression in macrophages derived from PBMCs and cultured with serum obtained from BC patients and healthy donors. n = 3 cell samples. K) The schematic diagram illustrated that CTH was the dominant H_2_S synthesizing enzyme in BC tissues and high CTH expression was significantly correlated with low infiltrations of M2 macrophages and prolonged survival of BC patients. Statistical significance was calculated using an unpaired t‐test in A, C, and D, Log‐rank (Mantel‐Cox) test in B and E, and one‐way ANOVA in G.

In mammals, H_2_S is mainly synthesized from cysteine or its derivatives by the enzymes CBS and CTH.^[^ ^6^ ^]^ Here, we first compared the mRNA levels of CBS and CTH using the TCGA‐BC database and found that the mRNA levels of CTH were significantly more abundant than those of CBS (*P* < 0.0001, **Figure** **1**A), suggesting that CTH might be the dominant H_2_S synthesizing enzyme in BC tissues. Moreover, compared to the low expression groups, patients with BC exhibiting elevated levels of CTH expression had a more favorable prognosis in terms of RFS (P = 0.053, Figure 1B) and DMFS (P = 0.053, Figure 1B). Nevertheless, CBS was not significantly correlated with the prognosis of patients with BC (P > 0.05, Figure 1B).

Given that H_2_S is an endogenous regulator of the immune system,^[^ ^21^, ^22^ ^]^ we sought to explore the correlation between CTH as an H_2_S synthesizing enzyme and immune cell infiltration. The CIBERSORT algorithm was used to assess the presence of immune cell infiltration within BC tissues profiled from TCGA‐BC. There was a notable disparity in the infiltration of B cells, plasma cells, Treg cells, monocytes, mast cells, and M2 macrophages observed between the groups characterized by high and low expression of CTH (*P* < 0.05, Figure 1C). Moreover, among all immune cells, M2 macrophages accounted for the largest concentrations, and CTH expression was significantly negatively correlated with the infiltration of M2 macrophages (Figure 1C). To verify these results, we further used the QUANTISEQ algorithm and found that decreased infiltration of M2 macrophages was related to elevated CTH expression levels (P = 0.003, Figure 1D), and M2 macrophages accounted for the largest concentrations, which were consistent with our previous findings. Subsequently, we divided the patients according to the expression levels of CTH and pro‐inflammatory factor IL‐1β. Kaplan–Meier curves showed that in the groups with high CTH expression, patients with high IL‐1β expression had shorter survival than those with low IL‐1β expression (P = 0.037, Figure 1E). However, in the group with low CTH expression, the levels of IL‐1β expression had no obvious correlation with the survival of patients with BC (P > 0.05, Figure 1E), which once more demonstrated a significant correlation between the expression of CTH and the prognosis of patients with BC.

To experimentally verify the above results, THP‐1 and RAW264.7 cells were chosen as cell models. In vitro, M0, M1, and M2 phenotype macrophages were successfully differentiated based on THP‐1 (Figure 1F; Figure S1A, Supporting Information) and RAW264.7 cells (Figure 1F; Figure S1B, Supporting Information). The amount of H_2_S released by TAM‐M1 macrophages was significantly greater than that released by TAM‐M2 macrophages (P < 0.001, Figure 1G). Moreover, increased expression of CTH was observed in TAM‐M1 macrophages compared to that in TAM‐M2 macrophages (Figure 1H). However, CBS did not detect significant differences between TAM‐M1 and TAM‐M2 macrophages (Figure 1H). In terms of mRNA levels, CTH also showed a stable increase in TAM‐M1 macrophages compared to TAM‐M2 macrophages (*P* < 0.001, Figure S1C, Supporting Information), while CBS did not (P > 0.05, Figure S1C, Supporting Information). To create TAMs in vitro that more closely resemble those in vivo, we further cultured macrophages with serum obtained from healthy donors or patients with BC. After 48 h of treatment, macrophages cultured with serum derived from patients with BC had lower CTH expression (P = 0.0135, Figure 1I; Figure S1D, Supporting Information) and higher CD206 expression (P = 0.0035, Figure 1I; Figure S1D, Supporting Information) than those cultured with serum derived from healthy donors. To verify these findings, macrophages induced from PBMCs of healthy donors were cultured with serum derived from healthy donors or patients with BC for 48 h. FCM analysis indicated that CTH and CD86 expression in macrophages cultured with serum derived from patients with BC was significantly decreased compared to that in macrophages cultured with serum derived from healthy donors, whereas CD206 expression was increased (*P* < 0.001, Figure 1J; Figure S1E, Supporting Information).

These results suggested that CTH is the dominant H_2_S synthesizing enzyme in BC tissues and high CTH expression was significantly correlated with low M2 macrophage infiltration and prolonged survival in patients with BC (Figure 1K).

### 2.2 H_2_S Promotes TAM‐M1 Polarization In Vitro

We further investigated the effect of increasing doses of NaHS, an H_2_S donor, on TAM polarization, and the results indicated that NaHS at a dose of 50 or 100 µm showed no marked influence on the expression of CD86 as an M1 macrophage marker and weakly decreased the expression of CD206 as an M2 macrophage marker (Figure S2A,B, Supporting Information). NaHS at a dose of 200 µm weakly promoted TAM‐M1 polarization (Figure S2A,B, Supporting Information). Surprisingly, NaHS at 400 µm concentration significantly increased the expression of CD86 and inhibited the expression of CD206, and NaHS then promoted TAM‐M1 polarization in a dose‐dependent manner (Figure S2A,B, Supporting Information). The mean maximal half‐effective concentration (EC50) of NaHS in CD86 was 387.8 µm (Figure S2C, Supporting Information). These results indicate that increased H2S levels are associated with TAM‐M1 polarization, and according to the EC50, 400 µm was chosen as the NaHS concentration in this study. We next analyzed the H_2_S levels in the supernatants collected from THP‐1 cells after adding increasing doses of NaHS for 6 h. The results indicated that NaHS increased the H_2_S levels in a dose‐dependent manner, indicating an increased endogenous production of H_2_S in the THP‐1 cell line culture system (Figure S2D, Supporting Information).

**Figure 2:**
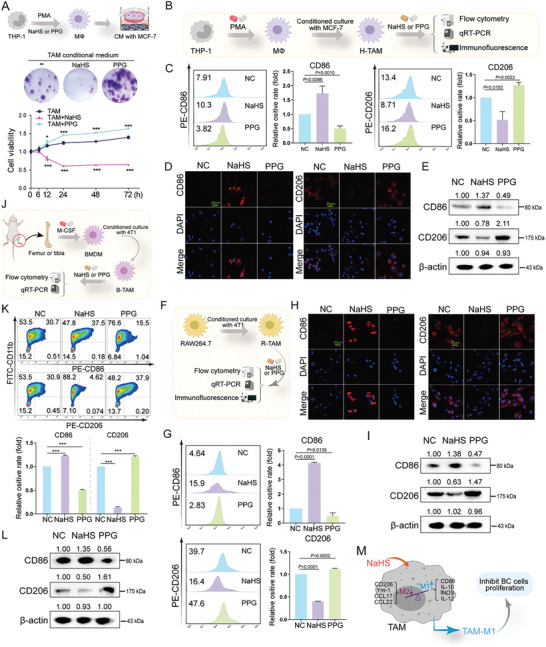
H_2_S promoted TAM‐M1 polarization and halted the proliferation, invasion, and migration of breast cancer cells in vitro. A) CCK8 assay was performed to identify the cell viability upon NaHS or PPG‐treated TAMs in MCF‐7 cells. B) Flowchart for constructing conditioned culture system of macrophages derived from THP‐1 and MCF‐7 cells. Flow cytometry analysis (C) and immunofluorescence analysis (D) of the expression levels of CD86 as M1 macrophage marker and CD206 as M2 macrophage marker in H‐TAM received NaHS or PPG treatment. n = 3 cell samples. E) Real‐time PCR analysis of the expression of IL‐1β and IL‐12 (as M1 macrophages marker), CCL17, and CCL22 (as M2 macrophages marker), with β‐actin serving as an internal control, in H‐TAM received NaHS or PPG treatment. n = 3 cell samples. F) Flowchart for constructing conditioned culture system of macrophages derived from RAW264.7 and 4T1 cells. Flow cytometry analysis (G) and immunofluorescence analysis (H) of the expression levels of CD86 as M1 macrophage marker and CD206 as M2 macrophage marker in H‐TAM received NaHS or PPG treatment. n = 3 cell samples. I) Real‐time PCR analysis of the expression of iNOS and IL‐1β (as M1 macrophages marker), Ym‐1 and Arg‐1 (as M2 macrophages marker), with β‐actin serving as an internal control, in R‐TAM received NaHS or PPG treatment. n = 3 cell samples. J) Flowchart for constructing conditioned culture system of macrophages derived from BMDMs and 4T1 cells. K) Flow cytometry analysis of the expression levels of CD86 as M1 macrophage marker and CD206 as M2 macrophage marker in H‐TAM received NaHS or PPG treatment. n = 3 cell samples. L) Real‐time PCR analysis of the expression of iNOS and IL‐1β (as M1 macrophages marker), Ym‐1 and Arg‐1 (as M2 macrophages marker), with β‐actin serving as an internal control, in B‐TAM received NaHS or PPG treatment. n = 3 cell samples. M) The schematic diagram illustrated that both endogenous and exogenous H_2_S could promote TAMs repolarization to the M1 phenotype in BC. Statistical significance was calculated using one‐way ANOVA in A, C, G, and K; ^*^ *p* < 0.05, ^**^ *p* < 0.01, ^***^ *p* < 0.001.

To examine the impact of endogenous and exogenous H_2_S in TAMs on BC cell proliferation, we used DL‐propargylglycine (PPG) as a CTH inhibitor (Figure S2A, Supporting Information) and NaHS as an exogenous H_2_S donor. The cell viability assay indicated that NaHS‐treated TAMs significantly inhibited the proliferation of MCF‐7 cells compared to the untreated TAMs groups, whereas PPG‐treated TAMs were beneficial to the proliferation of MCF‐7 cells (*P* < 0.0001, **Figure** **2**A).

To elucidate these findings, we measured the phenotypic changes in macrophages before and after treatment with NaHS or PPG. THP‐1, RAW264.7, and bone marrow primary macrophages (BMDMs) were selected as cellular models for the study. Then, we constructed a conditioned culture system of macrophages and BC cells. TAMs derived from THP‐1 cells (H‐TAMs) (Figure 2B), RAW264.7 cells (R‐TAMs) (Figure 2F), and BMDMs (B‐TAMs) (Figure 2J) were constructed. Exposure to NaHS led to a decrease in CD206 as an M2 macrophage marker and an increase in CD86 as an M1 macrophage marker in H‐TAMs (P < 0.05, Figure 2C; *P* < 0.05, Figure 2D; Figure S2B, Supporting Information), R‐TAM (P < 0.05, Figure 2G; *P* < 0.05, Figure 2H; Figure S2C, Supporting Information), and B‐TAM (*P* < 0.05, Figure 2K). However, cells subjected to PPG treatment exhibited elevated levels of CD206 expression and reduced levels of CD86 expression compared to the control groups (P < 0.05, Figure 2C,G,K). PCR analysis confirmed these findings at the mRNA levels (P < 0.05, Figure 2E,I,L).

These results indicate that both endogenous and exogenous H_2_S can promote TAM repolarization to the M1 phenotype in BC (Figure 2M).

### 2.3 H_2_S Restricts Tumor Growth in Response to TAM‐M1 Repolarization in BC‐Bearing Mice

**Figure 3:**
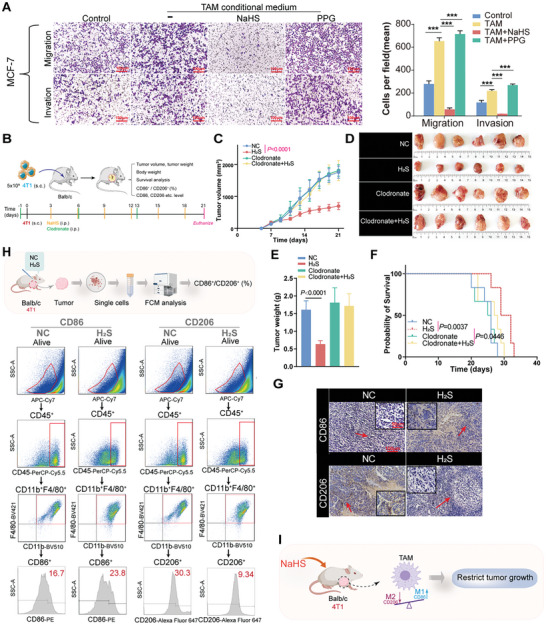
H_2_S prevented tumor growth in BC‐bearing mice. A) Trans‐well assays of the migration and invasion ability of MCF‐7 cells received various treatments. B) Treatment overview of 4‐week‐old female BALB/c mice inoculated with subcutaneous 4T1‐ tumors. C) Tumor growth curves of different groups of mice. D) Representative images of tumors from four groups of mice after being euthanized at the endpoint of the study. E) Tumor weight was measured from four groups of mice after being euthanized at the endpoint of the study. F) Kaplan–Meier survival curves for four groups of mice that received various treatments. G) Representative immunostaining of CD86 and CD206 in tumor tissues from four groups of mice based on polychromatic immunohistochemistry analysis (× 200 magnification and × 400 magnification). H) Flow cytometry analysis of CD86 and CD206 expression in CD45^+^F4/80^+^CD11b^+^ macrophages in tumors collected from mice received normal saline or NaHS treatment. I) The schematic diagram illustrated that H_2_S prevented tumor growth in BC‐bearing mice, and the promotion of TAM‐M1 repolarization was the key link for exerting this effect. n = 6 mice. Statistical significance was calculated using one‐way ANOVA in A, C, and E, and the Log‐rank (Mantel‐Cox) test in F. ^*^ *p* < 0.05, ^**^ *p* < 0.01, ^***^ *p* < 0.001.

Typically, TAMs can change their phenotypes, depending on the signals from the surrounding microenvironment, and can either kill tumor cells or promote tumor cell growth and metastasis.^[^ ^23^ ^]^ Therefore, due to the well‐established roles of H2S in TAM repolarization, we sought to demonstrate whether H2S inhibited BC progression. It was found that conditioned cultured with TAMs enhanced the invasion ability and migration rate of MCF‐7 cells compared to the control group (*P* < 0.0001, **Figure** **3**A), which was consistent with previous findings that TAMs promote metastasis of BC.^[^ ^24^ ^]^ Moreover, NaHS significantly weakened the invasion ability and migration rate of MCF‐7 cells compared to the TAM group (P < 0.0001, Figure 3A). However, MCF‐7 cells treated with PPG exhibited enhanced invasion ability and migration rate compared to the TAM group (*P* < 0.0001, Figure 3A). These results show that both endogenous H_2_S and exogenous H_2_S are harmful to the invasion and migration of BC in vitro.

To verify whether the inhibitory effect of H_2_S on BC cell proliferation and metastasis applies to different subtypes of BC cells, we performed colony formation and Transwell assays using the MDA‐MB‐231 cell line. The results of the colony formation assays indicated that in the conditioned culture system based on TAM supernatant, NaHS treatment significantly abrogated MDA‐MB‐231 cell proliferation compared to phosphate buffered saline (PBS) treatment (*P* < 0.0001, Figure S3A, Supporting Information). In contrast, PPG administration markedly promoted MDA‐MB‐231 cell proliferation compared to PBS treatment (P = 0.0005, Figure S3A, Supporting Information). The results of the Transwell assay also revealed that the migration and invasion of MDA‐MB‐231 cells were suppressed or enhanced by NaHS (migration: *P* < 0.0001, Figure S3B, Supporting Information; invasion: *P* < 0.0001, Figure S3B, Supporting Information) or PPG (migration: P = 0.0083, Figure S3B, Supporting Information; invasion: P = 0.0396, Figure S3B, Supporting Information) treatment, respectively. Collectively, these findings demonstrate that the inhibitory effect of H_2_S on tumor proliferation and metastasis is not limited to BC subtypes.

Given the observed relationship between H_2_S and TAM repolarization, as well as the detrimental impact of H_2_S on the proliferation, invasion, and migration of BC cells in laboratory settings, it is plausible that H_2_S may exhibit therapeutic efficacy in an in vivo context. Therefore, an in vivo antitumor assay of H_2_S donor NaHS was conducted using 4T1 BC‐bearing mice, with clodronate liposomes used as macrophage scavengers. Mice were treated as described in Figure 3B. After treatment, NaHS significantly reduced the tumor volume (*P* < 0.0001, Figure 3C,D) and tumor weight (*P* < 0.0001, Figure 3E) and prolonged survival (P = 0.0037, Figure 3F), compared to the NC groups, while these effects were disrupted by clodronate liposomes (P > 0.05, Figure 3C–F). Meanwhile, there were no notable variations in body weight across the different groups (Figure S3A, Supporting Information). These results showed that H_2_S inhibited BC tumor growth depending on the presence of macrophages.

To further investigate the potential correlation between the suppression of tumor growth in BC‐bearing mice induced by H_2_S and TAM repolarization, we assessed the levels of CD86 and CD206 expression in macrophages isolated from the tumor tissues of BC‐bearing mice. In accordance with the in vitro results, it was noted that H_2_S decreased the expression of CD206 and upregulated the expression of CD86 in the macrophages of BC‐bearing mice (CD86, P = 0.0001, CD206, *P* < 0.0001, Figure 3H; Figure S3B, Supporting Information). Moreover, we further immunostained tumor tissues with CD86 and CD206, and the results were fully consistent with our previous findings that treatment with NaHS led to a decrease in CD206 and an increase in CD86 in tumor tissues obtained from BC‐bearing mice (Figure 3G). Next, to explore the proliferation and metastasis of cancer cells in BC‐bearing mice, we also immunostained tumor tissues with the proliferation marker Ki67 and the epithelial‐mesenchymal transition markers E‐Cadherin and Vimentin. The results revealed that NaHS significantly decreased the expression of Ki67, E‐Cadherin, and Vimentin in tumor tissues obtained from BC‐bearing mice compared to the NC groups, but these effects were abrogated by clodronate liposomes (Figure S3C, Supporting Information).

These findings suggested that H_2_S prevented tumor growth in BC‐bearing mice and that the promotion of TAM‐M1 repolarization is a key link for exerting this effect (Figure 3I).

Meanwhile, a safety assessment of H_2_S in mice showed that no mice died in either experimental group. A blood test on day 14 post‐injection of NaHS showed no significant difference in the ratio of cell subpopulations between mice and control (PBS) animals (P > 0.05, Figure S4A, Supporting Information). Additionally, no significant changes in the relative weight of the body or organs, including the heart, lungs, liver, spleen, or stomach, were observed in either male or female mice after injection of NaHS solution compared to the control (PBS) mice (P > 0.05, Figure S4B–G, Supporting Information). Histological evaluations of the heart, lungs, liver, spleen, and colon in both males and females after i.p. injection of NaHS solution revealed no pathological changes compared to the findings in control (PBS) animals (Figure S4H, Supporting Information). In summary, the therapeutic dose of NaHS solution caused no abnormal changes in body weight, blood profiles, or histopathology, indicating the safety of therapeutic doses of H_2_S in mice.

### 2.4 H_2_S‐Mediated GRP78 S‐Sulfhydration Activates the IRE‐1α‐ERS Pathway to Promote TAM‐M1 Polarization

**Figure 4:**
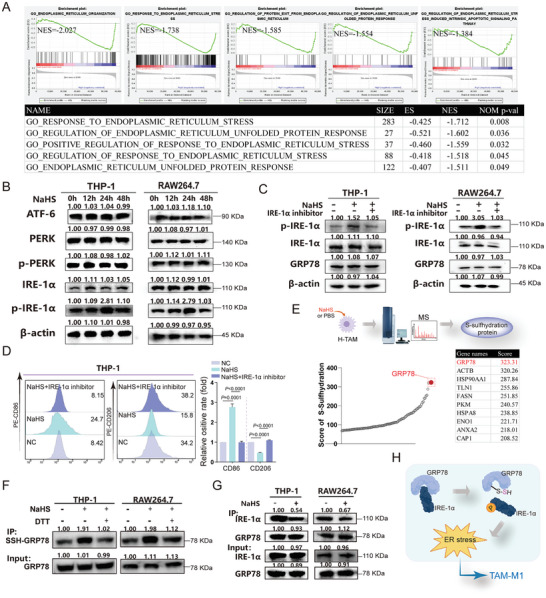
H_2_S induced the activation of the IRE‐1α/ERS pathway via S‐Sulfhydrylatiing GRP78 in BC‐TAMs. A) GSEA analysis based on the expression levels of CTH using 1091 breast cancer patients from the TCGA‐BC database. B) Western blotting images of PERK, p‐PERK, IRE‐1α, p‐IRE‐1α, and ATF6 protein levels in H‐TAM and R‐TAM received NaHS treatment in 0 h, 12 h, 24 h, and 48 h, respectively. n = 3 cell samples. C) The protein levels of IRE‐1α, p‐IRE‐1α, and GRP78 in H‐TAM (left) and R‐TAM (right) suffer to 4µ8C (32 µM) as a selective IRE1 Rnase inhibitor. n = 3 cell samples. D) Flow cytometry analysis of the expression levels of CD86 and CD206 in H‐TAM received NaHS or 4µ8C treatment (32 µM). n = 3 cell samples. E) Summarized data of proteins S‐sulfhydration in macrophages treated with NaHS. S‐Sulfhydrylated proteins of macrophages treated with NaHS were purified by biotin switch, followed by MS analysis. n = 3 cell samples. F) GRP78 S‐sulfhydration of macrophages received NaHS with or without dithiothreitol (DTT, 1 mmol L^−1^ for 1 h) was detected by a biotin switch. n = 3 cell samples. G) Western blots showing IRE‐1α and GRP78 interactions in H‐TAM and R‐TAM after NaHS administration. n = 3 cell samples. H) The schematic diagram illustrated that H_2_S S‐sulfhydrated GRP78, induces IRE‐1α dissociation from GRP78, which enhances the phosphatase activity of IRE‐1α itself and repolarizes TAMs into M1 phenotype. Statistical significance was calculated using one‐way ANOVA in D.

Next, to explore the underlying mechanism of H_2_S promoting TAM‐M1, we used the gene expression data of 1091 patients with BC from the TCGA‐BC database and divided the patients into high and low groups according to the expression levels of CTH. GSEA showed that the increase in CTH expression was associated with ER assembly, ER response stress, regulation of ER protein excretion, regulation of ER unfolded protein response, and regulation of endogenous apoptosis signaling pathways induced by ER stress (**Figure** **4**A). These results suggest that H_2_S promotes TAM‐M1 polarization via the ERS pathway. Mammalian cells contain three ER transmembrane proteins, namely ATF6, IRE1α, and PERK, which function as sensors of the ER.^[^ ^16^ ^]^ Therefore, to explore whether H_2_S can activate the ERS pathway, western blot analysis was used to evaluate the expression of PERK, p‐PERK, IRE‐1α, p‐IRE‐1α, and ATF6 in H‐TAMs and R‐TAMs treated with NaHS at 0, 12, 24, and 48 h. The findings showed no significant alteration in the expression of PERK, p‐PERK, IRE‐1α, and ATF6 after H_2_S treatment but a significant upregulation in p‐IRE‐1α expression both in H‐TAMs and R‐TAMs at 24 h (Figure 4B). These results suggested that H2S did not affect the expression of IRE‐1α, but activated it, reaching its peak at 24 h. Moreover, the increase in p‐IRE‐1α expression induced by H_2_S was significantly reversed by an IRE‐1α activation inhibitor, whereas the expression of IRE‐1α did not change by 4µ8C (32 µm) as a selective IRE1 Rnase inhibitor (Figure 4C). Subsequently, FCM analysis indicated that exposure to NaHS led to a decrease in CD206 and an increase in CD86 in macrophages, which were abrogated by 4µ 8C (Figure 4D).

Growing evidence suggests that S‐sulfhydration, as a form of PTM, plays a significant role in the physiological effects exerted by H_2_S.^[^ ^25^, ^26^, ^27^ ^]^ We first evaluated the total S‐sulfhydration in macrophages after NaHS treatment. The results of mass spectrometry (MS) showed that after NaHS treatment, 973 proteins were S‐sulfhydrated by H2S, among which the GRP78 S‐sulfhydration level was the most notably increased in macrophages that received NaHS treatment (score = 323.1, Figure 4E). To verify these results, macrophages were treated with dithiothreitol (DTT) at a concentration of 1 mmol L^−1^ for 1 h to create a reduced environment. The results showed that although H_2_S increased the S‐sulfhydration of GRP78, this modification was significantly reduced by treatment with DTT, suggesting the release of the sulfhydryl (‐SH) group from the critical cysteine residue in GRP78 (Figure 4F).

It has been reported that GRP78, a master chaperone protein, plays a critical role in maintaining ER homeostasis. When GRP78 binds to IRE‐1α, the ER remains inactive. When GRP78 dissociates from IRE‐1α, IRE‐1α is phosphorylated as an active state, which subsequently induces ERS.^[^ ^16^ ^]^ Therefore, co‐immunoprecipitation analysis was conducted to investigate how GRP78 S‐sulfhydration activates the IRE‐1α pathway. The results revealed an interaction between GRP78 and IRE‐1α, which was prevented by NaHS as an H_2_S donor (Figure 4G). Meanwhile, NaHS treatment did not alter GRP78 expression (Figure 4C). The findings demonstrated a notable inhibition of the binding of GRP78 with IRE‐1α in the presence of H2S.

These findings suggested that H_2_S S‐sulfhydrated GRP78, induces IRE‐1α dissociation from GRP78, which enhances the phosphatase activity of IRE‐1α itself and repolarizes TAMs into the M1 phenotype (Figure 4H).

### 2.5 Cys420 Mutation Blocks GRP78 S‐Sulfhydration and TAM‐M1 Repolarization

**Figure 5:**
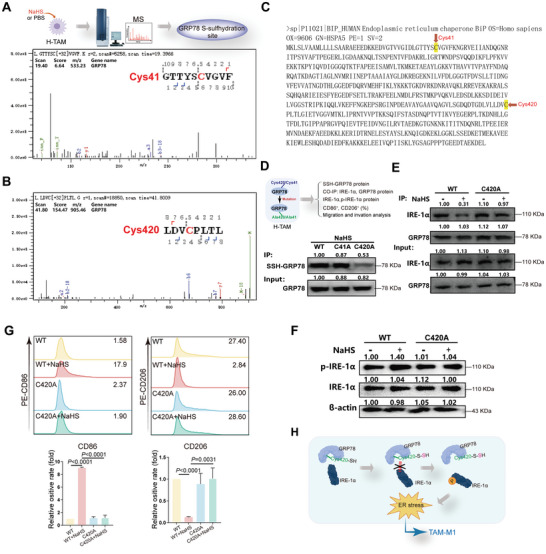
S‐sulfhydration of GRP78 was determined at cysteine 420. Scheme for examining the target cysteine residue for S‐sulfhydration on GRP78 (up). MS showed the cysteine 41 (A) and cysteine 420 (B) residue S‐sulfhydrated by H_2_S on GRP78. S‐Sulfhydrylated GRP78 of H‐TAM received NaHS administration was purified by biotin switch, followed by MS analysis. C) GRP78 protein sequence diagram. The arrows in the figure indicated the Cys41 and Cys420 points respectively. D) Western blotting images showing the S‐Sulfhydration of GRP78 of wild‐type (WT), Cys41, and Cys420 mutations in H‐TAM after NaHS treatment. GRP78 S‐sulfhydration was detected by a biotin switch. n = 3 cell samples. E) Cell lysates were immunoprecipitated with an anti‐GRP78 antibody, and the immunoprecipitated proteins were subjected to immunoblot analysis with anti‐IRE‐1α antibodies. The total lysates were analyzed with anti‐GRP78, anti‐IRE‐1α, and anti‐β‐actin antibodies in WT or Cys420 mutation H‐TAM with or without NaHS incubation. n = 3 cell samples. F) Western blotting images showing the expression of IRE‐1α and p‐IRE‐1α in WT or Cys420 mutation H‐TAM with or without NaHS incubation. n = 3 cell samples. G) Flow cytometry analysis showing the effects of WT or Cys420 mutants on H_2_S‐induced TAM‐M1 repolarization. n = 3 cell samples. H) The schematic diagram illustrated that Cys420 was specifically S‐sulfhydrated and was a critical residue for TAMs phenotype repolarization. Statistical significance was calculated using one‐way ANOVA in G.

We then proceeded to conduct a more detailed investigation of the specific cysteine residues that are targeted for S‐sulfhydration in GRP78. The S‐sulfhydrated proteins were tagged as described in the Experimental section, followed by MS. The MS suggested that H_2_S might S‐sulfhydrate the GRP78 protein on the Cys41 (**Figure** **5**A,C) or Cys420 site (Figure 5B,C). Hence, to detect the S‐sulfhydrated cysteine residue, plasmids encoding GRP78 with mutations at Cys41 or Cys420 to alanine (C41A or C420A) or wild‐type (WT) plasmids were introduced into THP‐1 cells via transfection. The biotin‐switch assay indicated that treatment with NaHS at a concentration of 400 µm for 24 h enhanced the S‐sulfhydration of WT and C41A forms of GRP78, except for the C420A (Figure 5D). Moreover, co‐immunoprecipitation analysis indicated that after GRP78 mutation at Cys420, H_2_S failed to induce IRE‐1α dissociation from GRP78 (Figure 5E) and IRE‐1α phosphorylation (Figure 5F). These results indicate that Cys420 is an S‐sulfhydrated residue of GRP78.

To provide additional evidence of the significant involvement of GRP78 S‐sulfhydration at the Cys420 site in TAM repolarization, we evaluated the levels of CD86 and CD206 expression in TAMs with C420A using FCM. The results showed that following incubation with NaHS, the levels of CD86 and CD206 expression showed minimal changes in TAMs harboring the C420A mutation in the GRP78 (Figure 5G).

These data suggest that Cys420 is specifically S‐sulfhydrated and is a crucial residue for TAM phenotype repolarization (Figure 5H).

### 2.6 GRP78 S‐Sulfhydration at Cys420 Promotes TAM‐M1 Polarization and Inhibits Tumor Growth and Lung Metastasis in BC In Vivo

Our previous findings demonstrate that H_2_S inhibits BC invasion and migration by promoting TAM‐M1 polarization. Here, we further investigated whether GRP78 S‐sulfhydration at Cys420 also played a critical role in the inhibition of BC invasion and migration. Transwell analysis showed that C420A significantly abrogated the inhibitory effect of NaHS on tumor invasion and migration (Figure S5A, Supporting Information).

**Figure 6:**
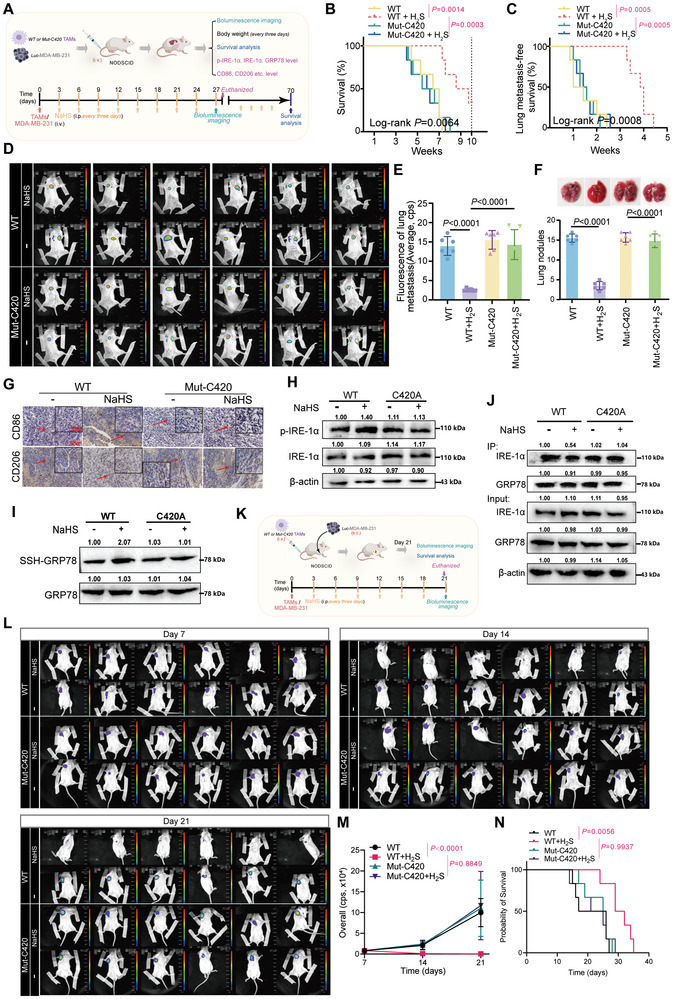
GRP78 Cys420 mutation eliminated the inhibitory effect of NaHS on tumor growth and lung metastasis of BC in vivo. A) Treatment overview of 6‐week‐old female NODSCID mice intravenously injected with MDA‐MB‐231‐Luc cells and WT or Mut‐Cys420 THP‐1 cells. Kaplan–Meier survival curves of overall survival (B) and lung metastasis‐free survival (C) for four groups of mice that received various treatments. D) Representative images of in vivo metastatic lungs of mice. E) Quantification of lung metastasis in four groups of mice. F) Number of lung metastase nodules of four groups of mice. G) Representative immunostaining of CD86 and CD206 in lung tissues from four groups of mice based on polychromatic immunohistochemistry analysis (× 200 magnification and × 400 magnification). H) Western blotting images showing the expression of IRE‐1α and p‐IRE‐1α in tissue samples of lung metastasis nodules from GRP78‐WT and GRP78‐C420A mice with or without NaHS treatment. I) Western blotting images showing the S‐Sulfhydration of GRP78 in tissue samples of lung metastasis nodules from GRP78‐WT and GRP78‐C420A mice with or without NaHS treatment. GRP78 S‐sulfhydration was detected by a biotin switch. J) Cell lysates were immunoprecipitated with an anti‐GRP78 antibody, and the immunoprecipitated proteins were subjected to immunoblot analysis with anti‐IRE‐1α antibodies. The total lysates were analyzed with anti‐GRP78, anti‐IRE‐1α, and anti‐β‐actin antibodies. K) Schematic diagram of the orthotopic xenograft breast cancer model based on NODSCID mice using MDA‐MB‐231 cell line. Specifically, 2 × 10^6^ per 100 µL MDA‐MB‐231 cells were injected into the right subaxillary of 4‐week‐old mice subcutaneously. After that, the mice were implanted with WT or GRP78‐Mut‐Cys420 THP‐1 cells with 5 × 10^6^ per 100 µL normal saline intravenously. NaHS saline solution was injected intraperitoneally (100 µmol per kg body weight) every three days for 3 weeks. L) Representative images of MDA‐MB‐231‐bearing mice that were implanted with the indicated THP‐1cells and treated with NaHS or PBS at the indicated times. n  =  6 animals per group. M) Relative change in the bioluminescence signal of intraperitoneal tumors in MDA‐MB‐231‐bearing mice in response to indicated treatment. N) Kaplan–Meier survival curves of mice that were received indicated treatment. n = 6 animals per group. Statistical significance was calculated using one‐way ANOVA in E, F, and M; the Log‐rank (Mantel‐Cox) test was used to analyze survival in B, C, and N.

To further investigate the impact of GRP78 S‐sulfhydration at the Cys420 residue on the metastasis of BC in vivo, WT or Mut‐Cys420 THP‐1 cells and MDA‐MB‐231 were intravenously injected into NODSCID mice, before treatment with either NaHS or PBS saline solution, as shown in **Figure** **6**A. After treatment, NaHS significantly prolonged overall survival (P = 0.0014, Figure 6B) and lung metastasis‐free survival (P = 0.0005, Figure 6C), reduced lung metastatic burden (*P* < 0.0001, Figure 6D,E), and reduced the number of lung metastasis nodules (*P* < 0.0001, Figure 6F) in the GRP78‐WT group, but this effect was eliminated in the GRP78‐C420A group (Figures 6B–F). Meanwhile, no notable variations in body weight were observed across the various groups of mice (P > 0.05, Figure S5B, Supporting Information). Moreover, further immunostained tumor tissues of lung metastasis nodules showed that treatment with NaHS led to a decrease in CD206 and an increase in CD86 in the GRP78‐WT group, but this effect was abrogated in the GRP78‐C420A group (Figure 6G).

Subsequently, tissues of lung metastasis nodules were taken for western blot analysis, and the results showed that consistent with our previous findings in vitro, in the GRP78‐C420A group, H_2_S failed to upregulate IRE‐1α phosphorylation (Figure 6H), S‐sulfhydrate GRP78 proteins (Figure 6I), and induce IRE‐1α dissociation from GRP78 (Figure 6J).

To further explore the therapeutic potential of GRP78 S‐sulfhydration at the Cys420 residue in suppressing tumor growth in vivo, we established an orthotopic cell line‐derived xenograft model using MDA‐MB‐231 cells. WT or GRP78‐Mut‐Cys420 THP‐1 cells were intravenously injected into NODSCID mice. The mice were subsequently treated with either NaHS or PBS saline solution, as shown in Figure 6K. Within 21 days after injection of MDA‐MB‐231 cells, mice treated with WT THP‐1 cells and NaHS treatment exhibited decreased tumor growth rates (*P* < 0.0001, Figure 6L, 6 m, Figure S5C, Supporting Information) and longer survival times (P = 0.0056, Figure 6N) compared to mice treated with WT THP‐1 cells and PBS. In contrast, in mice implanted with GRP78‐Mut‐Cys420 THP‐1 cells, NaHS treatment had no significant effect on tumor growth rates (P = 0.8849, Figure 6L, 6 m, Figure S5C, Supporting Information) or survival times (P = 0.9937, Figure 6N).

Collectively, these findings indicate that the suppression of GRP78 S‐sulfhydration through the Cys420 mutation hinders the antitumor and antimetastatic effects induced by H_2_S in BC‐bearing mice.

## 3 Discussion

The TME is generally linked to the advancement of malignancy and unfavorable outcomes in BC.^[^ ^28^ ^]^ Among the TME cells, TAMs are the main members, accounting for 30%–50% of the total.^[^ ^29^, ^30^ ^]^ TAMs are important tumor‐promoting cells in the TME and are associated with poor prognosis in BC.^[^ ^24^ ^]^ Typically, macrophage subtypes can be classified as either classically activated (M1) macrophages, which exhibit pro‐inflammatory properties along with antitumor effects, enabling them to effectively identify and eliminate cancer cells through cytotoxic and phagocytic mechanisms. Conversely, alternatively activated (M2) macrophages exhibit anti‐inflammatory activity and facilitate tissue regeneration and proliferation.^[^ ^31^ ^]^ TAMs can alter their phenotypes based on signals from their surrounding environment, which can result in either the destruction of tumor cells or the facilitation of tumor cell proliferation and spread.^[^ ^23^ ^]^ Therefore, repolarizing TAMs toward the M1 phenotype without causing significant side effects may have therapeutic potential against BC.^[^ ^32^ ^]^ Several preclinical trials have investigated potential drugs that regulate TAM polarization to achieve potent anti‐BC therapy. However, there remains an unmet clinical need for an effective drug‐repolarizing TAM phenotype for BC treatment.^[^ ^24^ ^]^

PTMs, including phosphorylation, acetylation, nitrosylation, and ubiquitination, represent crucial regulatory mechanisms for cellular proteins and play important roles in various biological processes.^[^ ^1^ ^]^ These modifications dictate the timing, strength, and location of specific physiological responses, allowing cells to swiftly and adaptively react to both external and internal stimuli.^[^ ^33^ ^]^ Protein S‐sulfhydration is a newly discovered PTM that is mediated by H_2_S.^[^ ^34^ ^]^ H2S‐induced S‐sulfhydration of PTP1B promotes PERK activity in response to ER stress.^[^ ^13^ ^]^ The intrinsic ERS response in cancer cells affects malignant progression by modulating the activity of immune cells present in the TME.^[^ ^35^, ^36^ ^]^ Meanwhile, considerable evidence has demonstrated that ERS activation induces M1‐type polarization in macrophages.^[^ ^14^, ^37^ ^]^ However, it is unknown whether protein S‐sulfhydration in the ERS response alters TAMs, and the underlying mechanism is incompletely understood.

In this study, we confirmed that both the endogenous CTH‐H_2_S axis and exogenous H_2_S polarized macrophages toward the M1 phenotype by S‐sulfhydrating GRP78 proteins via activating the IRE‐1α‐ERS pathway, thereby inhibiting the proliferation, migration, and invasion ability of BC cells. In vivo experiments also confirmed that exogenous H_2_S provoked the M2 to M1 phenotypic transformation through S‐sulfhydrating GRP78 proteins to trigger the IRE‐1α‐ERS pathway, and exogenous administration of H_2_S decreased tumor burden, inhibited metastasis, and increased survival in subcutaneous transplantation mouse models of BC.

Recent findings have confirmed that various H_2_S donors can halt the proliferation, migration, and invasion of BC cells,^[^ ^38^, ^39^, ^40^, ^41^, ^42^ ^]^ indicating that H_2_S donors and related compounds show promise in the advancement of BC inhibition. Although H_2_S donors release high concentrations of H2S, it remains unclear whether the remaining structural fragments after H_2_S generation are related to the potent antitumor activity. Therefore, the antitumor effect of H_2_S itself in BC and its precise mechanism remain to be clarified.

Therefore, in this study, we used NaHS as an exogenous H_2_S donor that did not generate byproducts during H_2_S release. Our results confirmed the anti‐cancer activity of H_2_S in BC. In other cancers, H_2_S has also shown promising potential for cancer treatment, such as pancreatic cancer,^[^ ^43^ ^]^ hepatocellular carcinoma^[^ ^44^ ^]^ and glioblastoma.^[^ ^45^ ^]^ However, Taohua Yue et al. proved that the CBS‐H_2_S axis creates colorectal cancer (CC)‐friendly immune microenvironment by downregulating the CD8+ T cell/Treg ratio.^[^ ^46^ ^]^ One potential explanation for this discrepancy might be that the CTH protein exceeds the CBS protein by ≈12‐fold in BC tissues, indicating that CTH is the dominant source of endogenous H_2_S in BC. This finding is consistent with a previous report that CTH is the primary enzyme responsible for H_2_S generation in mammalian cells.^[^ ^47^ ^]^ Moreover, CTH was decreased in BC tissues, whereas CBS was increased in CC tissues compared to their respective normal tissues.

Additionally, a previous study has shown that CTH is the primary enzyme responsible for generating endogenous H_2_S in macrophages, with CBS appearing to be non‐essential for H_2_S formation in macrophages.^[^ ^48^ ^]^ Another reason was that Hellmich et al. introduced a plausible bell‐shaped model to elucidate the complex impacts of H_2_S in the progression of cancer.^[^ ^49^ ^]^ Specifically, exposure to low H_2_S concentrations may promote cancer development, whereas prolonged or increased exposure to higher H_2_S concentrations may aid in tumor suppression. In CC tissues, H_2_S can also be produced exogenously from luminal bacteria, except endogenously, resulting in uncontrollable concentrations of H_2_S in tumor tissues. In contrast, in BC tissues, H_2_S is mainly produced endogenously.

In this study, we found that H_2_S supports M1 macrophage polarization in BC. However, the molecular mechanism underlying this effect is unclear. Previous studies have indicated that H_2_S activates intracellular signaling pathways dependent on the S‐sulfhydrylation of specific cysteine residues in target proteins.^[^ ^25^, ^26^, ^27^ ^]^ Specifically, the thiol functional group of active cysteine (‐SH) undergoes modification to ‐SSH through interaction with sulfur present in H_2_S.^[^ ^2^ ^]^ Therefore, in this study, we first assessed the total S‐sulfhydration of proteins in BC‐TAM after NaHS treatment using MS. We found that, in response to NaHS treatment, 973 proteins were S‐sulfhydrated by H2S, among which GRP78 S‐sulfhydration levels were the most notably increased in macrophages treated with NaHS. Furthermore, KEGG analysis revealed the enrichment of S‐sulfhydration proteins in many endoplasmic reticulum S‐related pathways. To further determine whether H_2_S could regulate ERS, we further analyzed PERK, IRE‐1α, and ATF‐6 activities related to ERS activation. We noted that H_2_S only increased the phosphorylation of IRE‐1α but did not change the expression level of IRE‐1α, p‐PERK, PERK, and ATF‐6. In addition, the favorable effect of H_2_S on M1 phenotype polarization can be abrogated by a selective IRE1 Rnase inhibitor. ERS triggered by the accumulation of misfolded proteins and cellular metabolic was identified as a critical switch commanding M1–M2 macrophage polarization.^[^ ^50^ ^]^ For example, GP96 as a myeloid ER resident promotes alcohol‐induced liver damage by inducing inflammatory responses (M1) in liver macrophages.^[^ ^51^ ^]^ Zhao et al. proved that the ERS inducer promotes M1 polarization and switches from M2 to M1 in BMDMs both in vivo and in vitro.^[^ ^52^ ^]^ These data are consistent with our findings that H_2_S promotes M1 macrophage polarization by activating the IRE‐1α‐ERS pathway in BC.

In cases of proteostasis, the molecular chaperone GRP78, which interacts with immunoglobulin proteins, forms a complex with IRE‐1α to regulate the ER and prevent its activation. During ERS, GRP78 dissociates from IRE‐1α because of its increased affinity to misfolded or unfolded proteins, which enables IRE‐1α activation and subsequent ERS induction.^[^ ^16^ ^]^ GRP78 exhibited the highest S‐sulfhydration levels among these proteins. The biotin‐switch analysis confirmed that H_2_S enhanced the S‐sulfhydration of GRP78 but did not significantly change the expression level. Additionally, we found that H_2_S S‐sulfhydration of GRP78 at cysteine 420 might initiate sequential steps in ERS. Specifically, GRP78 S‐sulfhydration caused IRE‐1α to separate from GRP78, enabling IRE‐1α phosphorylation and activating ERS. Blocking GRP78 S‐sulfhydration by mutating cysteine 420 abrogated the inhibitory effect of H_2_S on BC metastasis in metastasis mouse models of BC. Therefore, targeting GRP78 S‐sulfhydration could prevent the malignant progression of BC.

Although our study demonstrates the potential value of H_2_S in identifying the malignant progression of BC, there are inevitable constraints inherent in our research that must be duly recognized. First, the main challenge for gas molecules is how to release them quickly, stably, and controllably to a specified location. Therefore, for H_2_S therapy to be applied more effectively in clinical settings, it is necessary to develop a longer half‐life, higher bioavailability, and better‐targeting platforms for H_2_S delivery. Second, S‐sulfhydration frequently leads to a change in the protein's conformation, which can modify the enzyme activity or function of the protein, thereby acting as a significant switch or regulator.^[^ ^2^ ^]^ How GRP78 S‐sulfhydration at cysteine 420 changes the conformation that induces IRE‐1α to dissociate from GRP78 remains to be further determined. Third, previous research has shown that H_2_S can influence the release of nitric oxide (NO) and that they can mutually enhance the effectiveness of each other.^[^ ^53^ ^]^ Hence, it is pertinent to explore the potential interaction between various gases and their combined efficacy in BC therapy.

In conclusion, we demonstrated that GRP78 S‐sulfhydration, mediated by the endogenous CTH‐H_2_S axis or exogenous H2S, can promote M1 macrophage polarization via activating the IRE‐1α‐ERS pathway, resulting in inhibition of tumor growth and metastasis in BC. GRP78 S‐sulfhydration at Cys420 leads to the dissociation of IRE‐1α from GRP78, enabling IRE‐1α phosphorylation and activating ERS. Targeting GRP78 S‐sulfhydration may be a promising approach for patients with BC.

## 4 Experimental Section

### Cell Lines

The cell lines (THP‐1, RAW264.7, MCF‐7, 4T1, and MDA‐MB‐231) were purchased from the Cell Bank of the Chinese Academy (Shanghai, China). THP‐1 cells were cultured in RPMI‐1640. RAW264.7, MCF‐7, and 4T1 were cultured in DMEM. MDA‐MB‐231 was cultured in Leibovitz's L‐15. All cells were supplemented with 10% FBS and 100 IU mL^−1^ Penicillin–Streptomycin solution at 37 °C in 5% CO_2_.

### Harvesting Bone Marrow‐Derived Macrophages

Bone marrow‐derived macrophages (BMDM) were extracted from mouse femurs. ACK lysis (Lonza) was used to deplete red cells. The residual cells were rinsed with PBS and then resuspended in DMEM supplemented with 20% FBS and 20 nanograms per milliliter of murine Macrophage Colony‐Stimulating Factor (M‐CSF) from Peprotech. A fresh medium was introduced to replace half of the existing medium after 3 days. Subsequently, on the 7th day, the cells that contained over 80% of CD11b^+^ macrophages were harvested and identified as BMDM for further analysis.

### Mouse Models

The Ethics Committee of China Medical University approved the animal experiments, which were conducted in accordance with the guidelines set by the Institutional Animal Care and Use Committee of China Medical University (approval number: CMU20242079). All mice were obtained from Beijing Hfk Bioscience Co. Ltd and housed in a barrier facility on a 12 h light–12 h dark cycle. Mice were put to sleep once the tumor size exceeded or equaled 200 mm^2^. Kaplan–Meier estimates were utilized to plot survival curves, which were then compared through log‐rank analysis. The average ± SEM is used to express all results. No mouse exhibited severe loss of body weight (>15%) or evidence of infections or wounds.

### Safety Assessment of H_2_S in Mice

6‐weeks‐old C57BL/6 mice were divided into two groups: Control and H_2_S. Each group had 10 mice, half male and half female. NaHS saline solution was injected intraperitoneally (100 µmol kg^−1^ body weight) every day for 14 days. PBS injection was used as a negative control. Mice were observed daily for general condition, body weights, food and water consumption, visible signs of intoxication, and animal death during drug treatment. On Day 14 post‐injection, the blood of each mouse was collected by cardio puncture, and a blood test was performed using a cell hematology analyzer (Nihon Kohden, Tokyo, Japan). All female and male mice in each group were euthanized in a CO_2_ chamber. Heart, lungs, liver, spleen, stomach, and colon were isolated and weighed at necropsy.

### Establishment of the Subcutaneous Xenograft Breast Cancer Model

In this work, two xenograft mouse models were established: a mouse BC cells 4T1‐derived xenograft model and a human BC cells MDA‐MB‐231‐derived xenograft model.

In the 4T1‐derived xenograft model, female BALB/c mice that were 4 weeks old were divided into four groups in a random manner: NC, H_2_S, Clodronate, and Clodronate combined with H_2_S. Clodronate liposomes (200 µL/mouse, Liposoma BV, Amsterdam, Netherlands) were intravenously injected 48 h before 4T1 injection to deplete macrophages in mice. 2 × 10^6^ per 100 µL 4T1 cells were injected subcutaneously into the right sub axillary of 4‐week‐old female BALB/c mice. NaHS saline solution was performed on the third day after injecting 4T1 cells, and injected intraperitoneally (100 µmol kg^−1^ body weight) every 3 days for 3 weeks. PBS injection was used as a negative control. Body weights and tumor volume were measured every 3 days. On Day 21 post‐ injection of 4T1 cells, mice were sacrificed, and tumor tissues were collected. The survival time of another batch of mice was monitored (n = 6/group).

In the MDA‐MB‐231‐derived xenograft model, female NODSCID mice that were 4 weeks old were injected with 2 × 10^6^ per 100 µL MDA‐MB‐231 cells into the right subaxillary subcutaneously. After that, the mice were implanted with WT or GRP78‐Mut‐Cys420 THP‐1 cells with 5 × 10^6^ per 100 µL normal saline intravenously. NaHS saline solution was injected intraperitoneally (100 µmol kg^−1^ body weight) every 3 days for 3 weeks. PBS injection was used as a negative control. Orthotopic xenograft bioluminescence was examined every week. After 3 weeks, mice bearing BC were killed. The survival time of another batch of mice was monitored (n = 6/group).

### Establishment of the Breast Cancer Lung Metastasis Model

To establish a lung metastasis model of BC, 5 × 10^5^ MDA‐MB‐231‐Luc cells per 100 µL normal saline injected intravenously into 6‐week‐old female NODSCID mice. Then, the mice were adoptive transfer of WT or Mut‐Cys420 THP‐1 cells with 5 × 10^6^ per 100 µL normal saline. After inoculating, the mice were administered an intraperitoneal injection of NaHS saline solution (100 µmol kg^−1^ body weight), every 3 days for 4 weeks. 4 weeks after cells injection, tumor lung metastasis was confirmed by real‐time bioluminescence imaging. Subsequently, the mice were euthanized. The lungs were promptly removed, cleansed, and preserved in Bouin's solution. The quantification of metastatic nodules present on the lung surface was conducted utilizing a dissecting microscope. The survival of the mice within 70 days was counted for survival analysis, and NaHS treatment was given every 3 days.

### H_2_S Measurement

The supernatants of cells were prepared in tightly sealed tubes on ice. 1% zinc acetate was added to entrap synthesized H_2_S at 37 °C. Additionally, 20 µm N, N‐dimethyl‐p‐phenylenediamine sulfate in 7.2 m HCl was added, followed by 30 µm FeCl_3_ in 1.2 m HCl. 10% trichloroacetic acid was subsequently added to stop the reaction. Protein was removed by adding 250 µL trichloroacetic acid and centrifugation at 14000 g for 5 min. The absorbance of the final solution was determined at 670 nm. The concentration of H_2_S in each reaction mixture was calculated against a calibration curve of NaHS.

### Biotin Switch Assay of S‐Sulfhydration

A biotin switch assay was conducted following the manufacturer's instructions. Briefly, cells or tumor tissues were disrupted in RIPA buffer supplemented with protease inhibitors. After that, the homogenized samples were sonicated and centrifuged at 13,000 g for 20 min at 4 °C, followed by BCA quantification of total protein. Then, 2 µg anti‐GRP78 antibody (Cat No: 11587‐1‐AP, Proteintech) and Protein A beads were sequentially added to the supernatant (1 mg mL^−1^) and incubated at 4 °C overnight. Next, the lysates were treated with HEN buffer containing 2.5% sodium dodecyl sulfate (SDS) and 20 mm methyl methanethiosulfonate (MMTS). The specimens were regularly agitated at a temperature of 50 °C for 30 min. Subsequently, acetone was employed to eliminate the MMTS. Subsequently, the proteins were precipitated at −20 °C for 1 h. Protein (20 µL) was aspirated as a control, and 4 mm N‐[6‐(biogenic amino)hexyl]− 3′‐(2‐pyridinedithio) propionic acid amide was added to the remaining protein. Finally, streptavidin‐agarose beads were applied to pull down the biotinylated proteins and eluted with SDS‐PAGE buffer, followed by Western blotting for protein quantification.

### Mass Spectrometry of S‐Sulfhydration Proteins

The proteins that were S‐sulfhydrated were labeled with biotin‐HPDP and purified using streptavidin‐agarose beads, as described above. They were then subjected to MS.

### Isolation of Tumor‐Infiltrating Macrophages

The tumor samples were sliced into small fragments using razors and subsequently exposed to a mixture consisting of collagenase IV (2.5 mg mL^−1^, Sigma) and DNase I (0.1 mg mL^−1^, Sigma) for a duration of 2 h. After that, the reaction was balanced out by adding 10% fetal bovine serum. The cells were washed using RPMI/ 2% FBS at room temperature by passing them through the strainer. The tissue homogenate was subjected to centrifugation using a discontinuous Percoll (Sigma‐Aldrich) gradient consisting of layers at 35% and 60% concentrations. Subsequently, individual cells were isolated from the interface for subsequent analysis.

### Flow Cytometry Analyses

Flow cytometry (FCM) analyses were performed on 2 million cells. Peripheral blood mononuclear cells (PBMCs) were stained with antibodies against CD14‐BV421 (Biolegend, US). BMDM were stained with antibodies against CD11b‐FITC (Biolegend, US). TAMs derived from tumor tissues were labeled with fixable viability dye‐APC‐Cy7, CD45‐Percp‐Cy5.5, CD11b‐BV510, and F4/80‐BV421 (Biolegend, US). CD206‐Alexa Fluor 647 and CD206‐PE were employed as M2 markers (Biolegend, US). CD86‐PE was employed as M1 markers (Biolegend, US). Unless otherwise specified, a dilution of 1: 200 was used for all antibodies conjugated with fluorochrome. After being washed once more, the cells were resuspended in 400 µL PBS before being collected and evaluated using a BD Fortessa flow cytometer.

### Western Blot Analysis

Membranes containing target proteins were cultured in primary antibody (CTH, CBS, β‐actin, GRP78, IRE‐1α, p‐IRE‐1α, ATF‐6, PERK, p‐PERK) overnight at 4 °C. β‐actin protein was used as a control. Blots obtained from western blot analysis were quantitated by ImageJ software (+/− standard error of the mean (SEM)).

### Plasmid Constructs and Transfection Cells

A GRP78 expression plasmid from SinoBiological (Cat: HG12063‐ACG) served as a template to generate site mutant construct according to the results of sulfhydration proteomics (GRP78C41A and GRP78C420A), generating a mutant substituting Ala for Cys residues at Cys41 or Cys420. Next, macrophages were transfected with these plasmids using GeneX Plus transfection reagent.

### Real‐Time PCR Quantification

The cells' total RNA was isolated utilizing TRIzol reagent (Thermo Fisher Scientific) following the manufacturer's instructions, with the inclusion of a DNase digestion step to remove any genomic DNA impurities. The RNA sample was subjected to reverse transcription following the guidelines provided by the manufacturers. Quantitative real‐time PCR (qPCR) was conducted utilizing the Fast SYBR Green PCR kit in conjunction with Applied Biosystems 7900HT Fast RT‐PCR System (Applied Biosystems). The quantity of RNA was determined using the ΔΔCT method. The data was standardized based on the expression of β‐actin and then contrasted with the control group.

### Transwell Migration and Invasion Assay

TAMs were exposed to a 400 µm NaHS saline solution for 24 h or treated with PPG, a CTH inhibitor, at a concentration of 10 mm for a period of 4 h. Then, MCF‐7 cells were conditioned cultured with these treated TAMs for 48 h.

### Cell Proliferation Assay and Colony Formation

Cell viability was measured with CCK8 assay (CCK‐8, Sigma–Aldrich). Cells were arranged in 96‐well cell culture plates with a concentration of 5000 cells per well and incubated with 400 µm NaHS saline solution for 24 h or PPG, a CTH inhibitor, at a concentration of 10 mm for a period of 4 h. Then, 100 µL of DMEM supplemented with 10% CCK8 was introduced to the cell culture and allowed to incubate for a duration of 2 h at 37 °C. The assessment of cellular viability was conducted through the quantification of the absorbance of the dye conversion product at a wavelength of 450 nm.

For colony formation assay, 400 treated cells per well were coated into 6‐well plates. After 14‐day incubation, these plates were washed with PBS twice, fixed with methanol for 10 min, and stained with 0.1% crystal violet solution within 10 min for further analysis.

### Immunohistochemistry

Immunohistochemistry was performed using tumors or lung tissues removed from mice. The tissues were fixed in 4% paraformaldehyde 48 h and then embedded in paraffin wax and cut into 4 mm serial sections. Next, endogenous peroxidases were quenched and the sections were washed three times with PBS. The sections were blocked with goat serum in PBS at 37 °C temperature for 30 min, then incubated with anti‐CD86 antibody (1:200, Abcam, ab270719, UK), anti‐CD206 antibody (1:200, Abcam, ab64693, UK), anti‐Ki67 antibody (1:200, Abcam, ab15580, UK), anti‐E‐Cadherin antibody (1:200, Abcam, ab231303, UK) and anti‐Vimentin antibody (1:200, Abcam, ab8978, UK) overnight at 4 °C separately. The sections were incubated with horseradish peroxidase‐conjugated secondary antibodies and subsequently treated with DAB (Dako, Carpinteria, CA, USA). Next the sections were subsequently stained with hematoxylin as a counterstain.

### Statistical Analysis

The expression profiles of mRNAs from TCGA were shown as raw data and each mRNA was normalized by log2 transformation for further analysis. All the data were presented as the mean ± standard errors of the means (SEMs). Results were harvested from a minimum of three separate trials and data shown were from representative experiments. Log‐rank (Mantel‐Cox) test was used to analyze survival. The statistical significance of differences between the two groups was analyzed by unpaired Student t‐test; one‐way analysis of variance (ANOVA) was employed for datasets comprising three or more groups, utilizing the GraphPad Prism Software and SPSS 19.0 software. Statistical significance was defined as having a *P*‐value less than 0.05.

### Compliance with Ethics Requirement

The Ethics Committee of China Medical University approved the animal experiments, which were conducted in accordance with the guidelines set by the Institutional Animal Care and Use Committee of China Medical University (approval number: CMU20242079).

## Conflict of Interest

The authors declare no conflict of interest.

## Author Contributions

Mingyi Ju and Weiwei Tong contributed equally to this work. Conceptualization, investigation, writing‐original draft: Mingyi Ju; validation, formal analysis, and visualization: Jia Bi, Xianxin Zeng, and Mingli Sun; data curation, methodology, and software: Aoshuang Qi; resources: Weiwei Tong and Jian Wen; supervision, writing – review & editing: Lin Zhao and Minjie Wei.

## Supporting information



Supporting Information

## Data Availability

The data that support the findings of this study are available in the supplementary material of this article.
