# Supplementary material for: Hydrogen Sulfide Promotes TAM‐M1 Polarization through Activating IRE‐1α Pathway via GRP78 S‐Sulfhydrylation to against Breast Cancer
Source: Adv Sci (Weinh). 2025 Jan 4;12(8):2413607. doi: 10.1002/advs.202413607 (PMC11848574; doi:10.1002/advs.202413607)

**
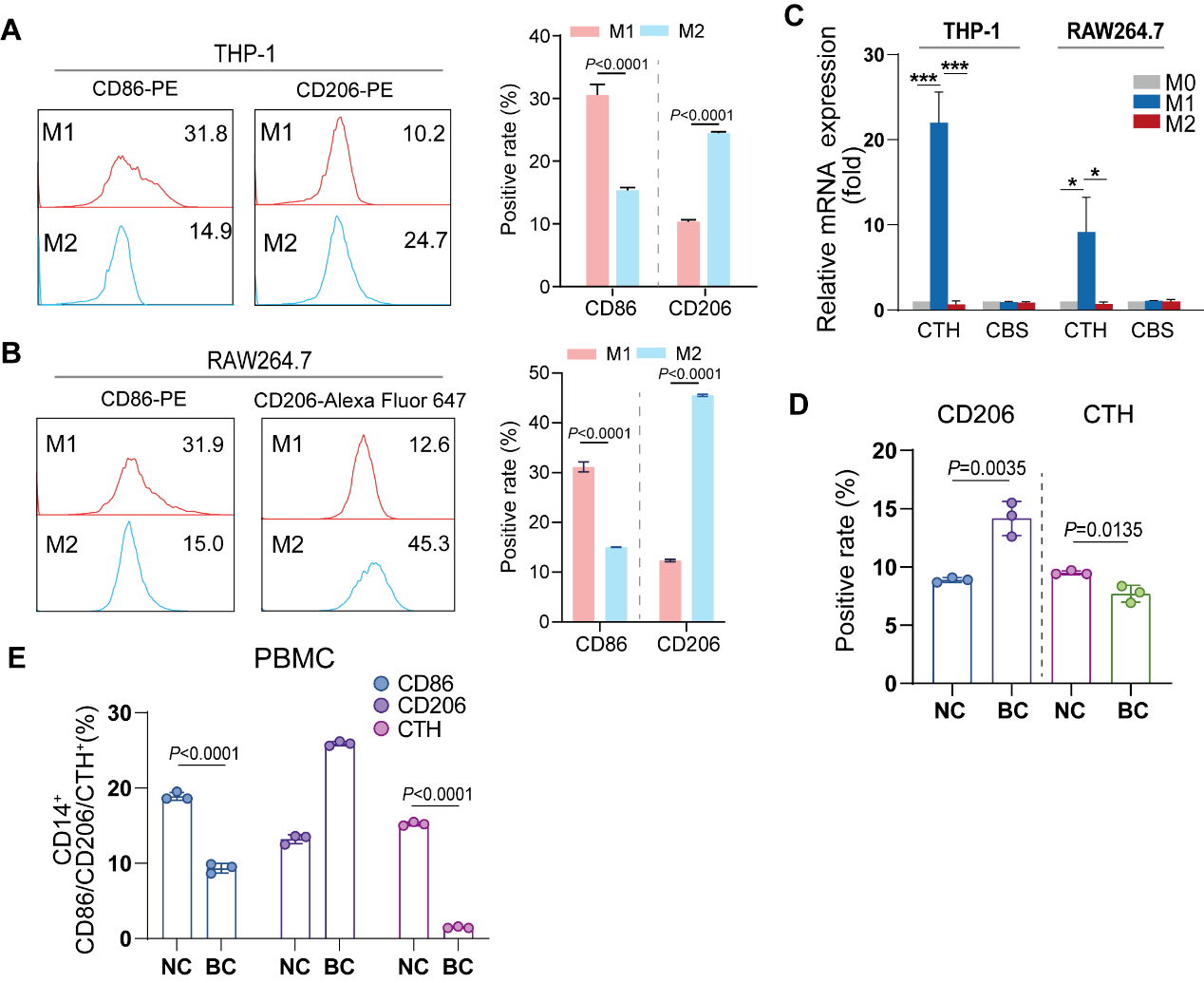
Figure S1. CTH, rather than CBS, was related to survival and TAMs polarization in BC.** M1 and M2 macrophages were successfully induced *in vitro* using THP-1 cells (A) and RAW264.7 (B) cells, confirmed by flow cytometry analysis. n = 3 cell samples. (C) mRNA expression levels of CTH and CBS in M0, M1 and M2 phenotypes macrophages derived from THP-1 and RAW264.7 cells were detected by Real-time PCR. n = 3 cell samples. (D) Quantitative analyses of CTH and CD206 expression in macrophages cultured with serum derived from BC patients and healthy donors. n = 3 cell samples. (E) Quantitative analyses of CTH, CD86 and CD206 expression in macrophages derived from PBMCs and cultured with serum obtained from BC patients and healthy donors. n = 3 cell samples. Statistical significance was calculated using an unpaired t test in A, B, D and E; one-way ANOVA in C. **p* < 0.05, ***p* < 0.01, ****p* < 0.001.


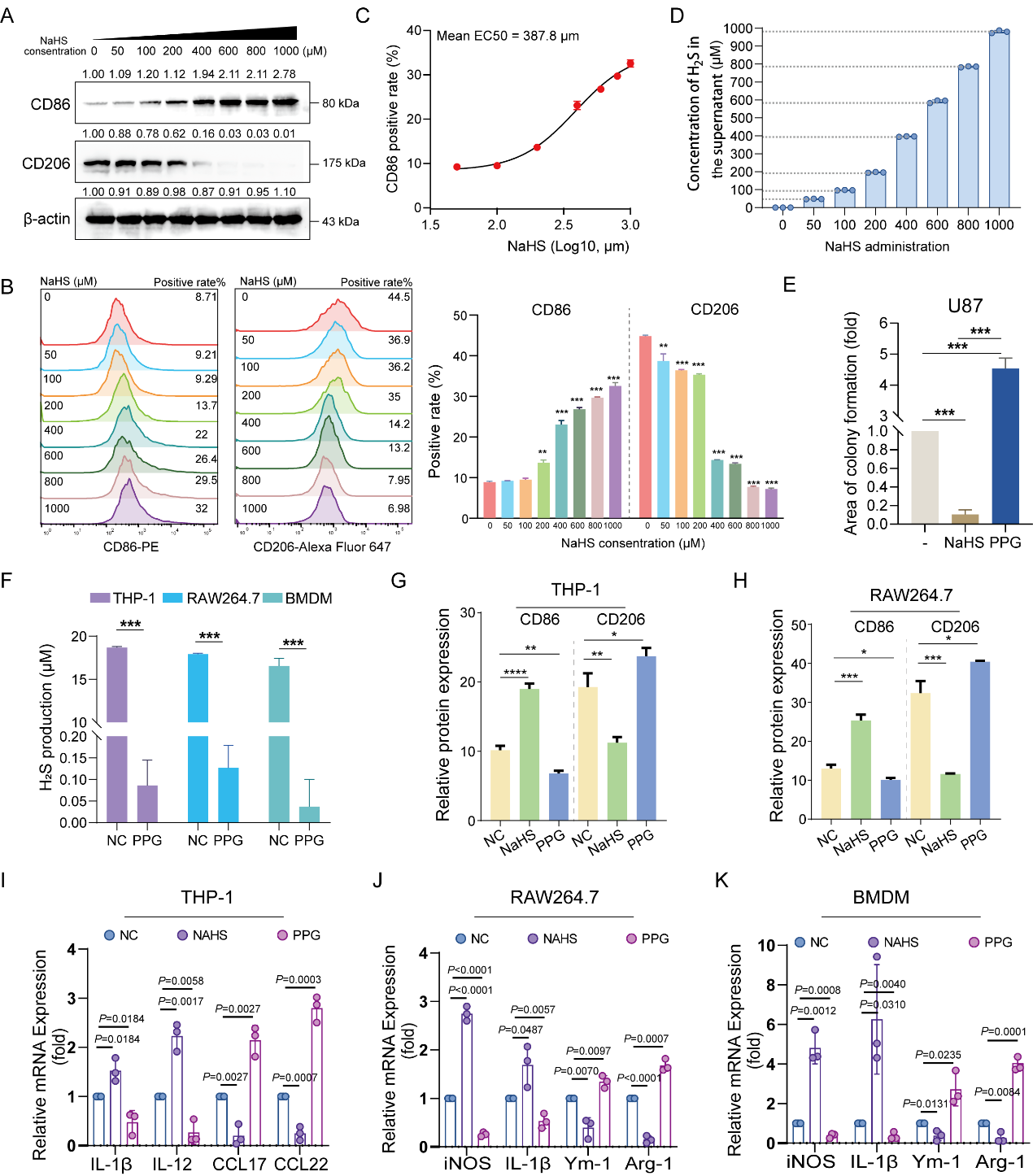


**Figure S2. H_2_S promoted TAM-M1 polarization *in vitro*.** H-TAM cells were treated with different concentrations (0–1000 μM) of NaHS for 24 h, protein expression of CD86 and CD206 were detected by western blot (A) and flow cytometry (B), respectively.** *0.01 <P* < 0.001, ****P* < 0.0001, compared with 0 μM NaHS, bars show the group mean ± SEM. (C) The effects of different concentrations (0–1000 μM) of NaHS on the expression of CD86, which was detected by flow cytometry. (D) The accumulation over 6 h of H_2_S in culture medium was measured in the experimental groups. (E) The proliferation capacities were detected by colony formation assay with bar charts showing colony number. (F) H_2_S production in THP-1, RAW264.7 and BMDM cells before or after PPG administration. The statistical analysis of the expression levels of CD86 as M1 macrophages marker and CD206 as M2 macrophages marker in THP-1 (G) and RAW264.7 cells (H) after NaHS or PPG administration, which is detected by flow cytometry analysis. The statistical analysis of the mRNA levels of the relative gene in THP-1 (I), RAW264.7 (J) and BMDM (K) cells received NaHS or PPG treatment, which is detected by real-time PCR analysis. n = 3 cell samples. Statistical significance was calculated using one-way ANOVA in B, E and G-K, and unpaired t test in F. **p* < 0.05, ***p* < 0.01, ****p* < 0.001.

**
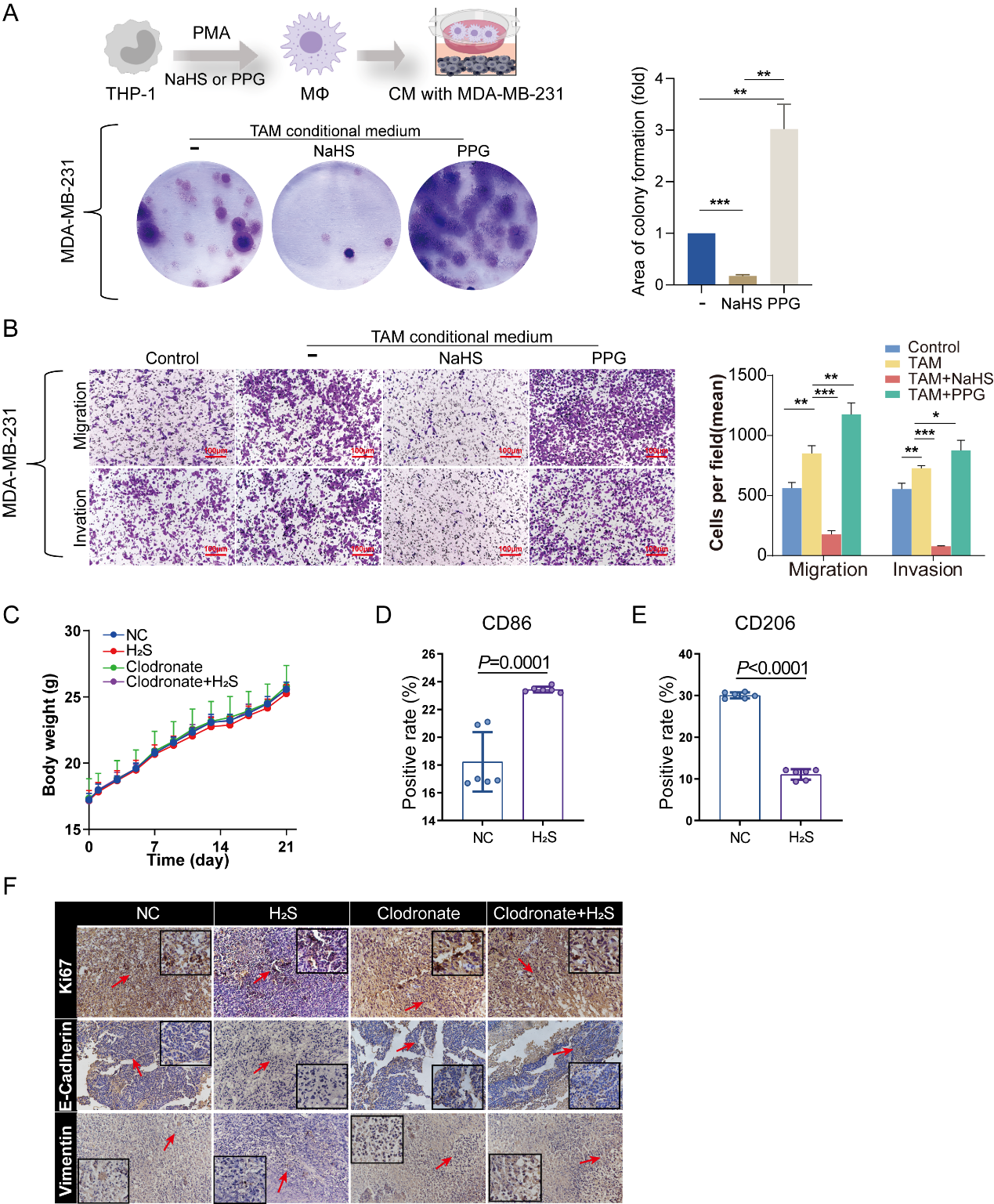
**

**Figure S3. H_2_S prevent MDA-MB-231 cell proliferation and invasion *in vivo* and *in vitro*.** MDA-MB-231 cells were incubated in a conditioned culture system using TAMs supernatant. (A) The proliferation capacities were detected by colony formation assay (left), with bar charts showing colony numbers (right). (B) Transwell assays were used to assess the invasion and migration of MDA-231 cells after treated with NaHS or PPG. Significance was determined by a two-sided, unpaired Wilcoxon rank-sum test. n.s. means no significant difference. (C) Body weight curves of different groups of mice. Quantitative analyses of CD86 (D) and CD206 (E) expression in CD45^+^F4/80^+^CD11b^+^ macrophages in tumors collected from mice received normal saline or NaHS treatment. (F) Representative immunostaining of Ki67, E-Cadherin and Vimentin in tumor tissues from four groups of mice based on polychromatic immunohistochemistry analysis (× 200 magnification and × 400 magnification). n = 3 cell samples. Statistical significance was calculated using one-way ANOVA in A, B and C, and an unpaired t test in D and E. **p* < 0.05, ***p* < 0.01, ****p* < 0.001.

**
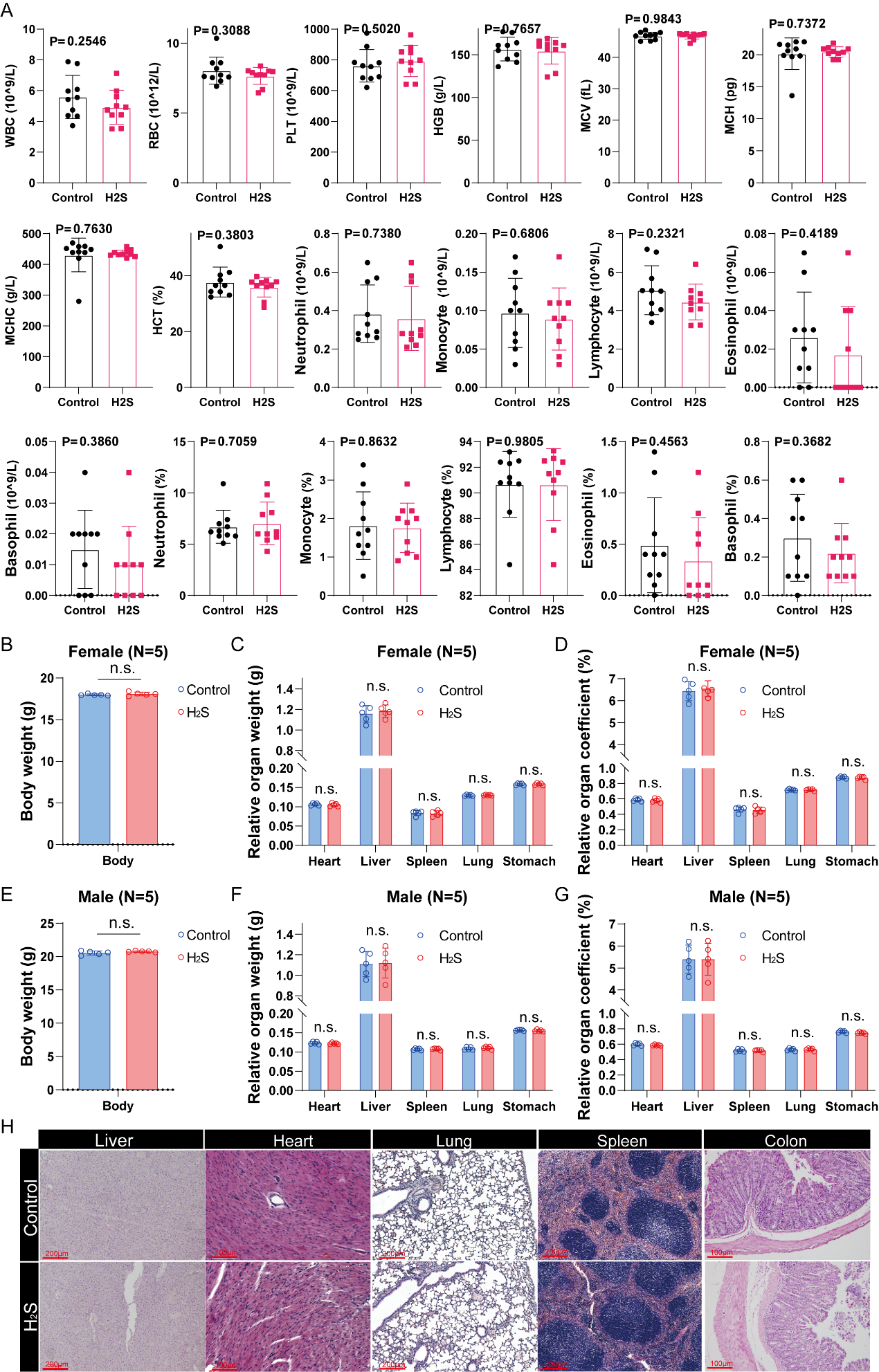
**

**Figure S4.** **Safety assessment of H**_2_**S in mice.** 6-weeks-old C57BL/6 mice were divided into two groups: Control and H_2_S. Each group had 10 mice, half male and half female. NaHS saline solution were injected intraperitoneally (100 µmol per kg body weight) every day for 14 days. PBS injection was used as a negative control. On Days 14 post-injection, blood of each mouse was collected by cardio puncture, and heart, lungs, liver, spleen, stomach and colon were isolated and weighted at necropsy. (A) The blood-routine-test of mice post-injection of PBS or NaHS saline solution on days 14. HGB, hemoglobin; HCT, hematocrit; MCH, mean corpuscular hemoglobin; MCHC, mean corpusular hemoglobin concentration; MCV, mean corpuscular volume; PLT, Platelets; RBC, red blood cell; WBC, white blood cell. The body weight (B), relative organ weight (C) and relative organ coefficient (D) of female mice post-injection of PBS or NaHS saline solution on days 14. The body weight (E), relative organ weight (F) and relative organ coefficient (G) of male mice post-injection of PBS or NaHS saline solution on days 14.$Organ coefficient=\frac{O\mathrm{rgan}\mathrm{weight}}{Body weight}\times100\%$ (H) The HE staining of multiple organs from mice injected with PBS or NaHS saline solution. Scale bar: 100 or 200 µm. Error bars indicated the standard deviation. Significance was determined by a two-sided, unpaired Wilcoxon rank-sum test. n.s. means no significant difference. Statistical significance was calculated using an unpaired t test in A-G. n.s. *P* > 0.05.

**
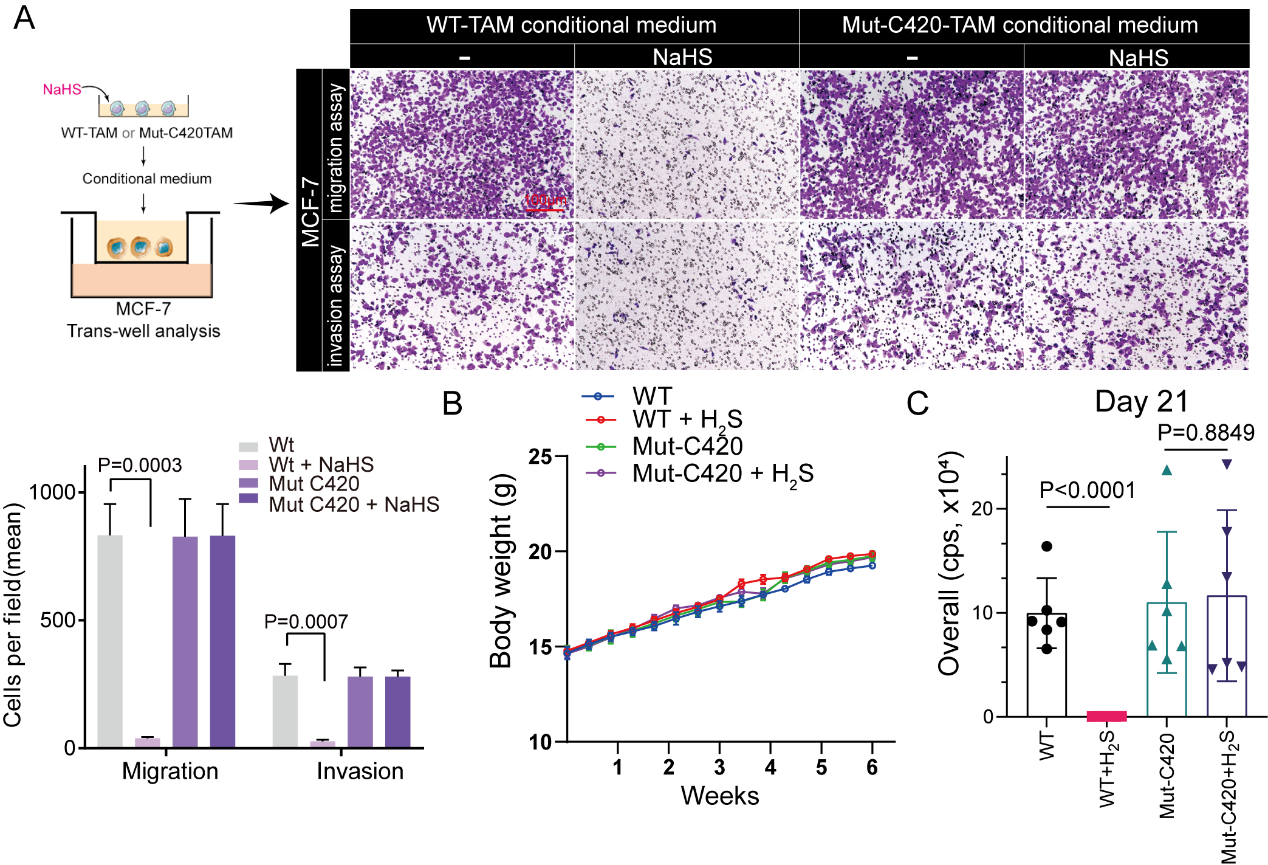
**

**Figure S5.** **GRP78 S-sulfhydration at Cys420 site of TAM halted lung metastasis in BC-bearing mice.** (A) Trans-well assays of the migration and invasion ability of MCF-7 cells received various treatment. (B) Body weight curves of four groups of mice. n = 6 mice. (C) Quantification of lung metastasis in four groups of mice in day 21. n = 6 mice. Statistical significance was calculated using one-way ANOVA in A, B and C.


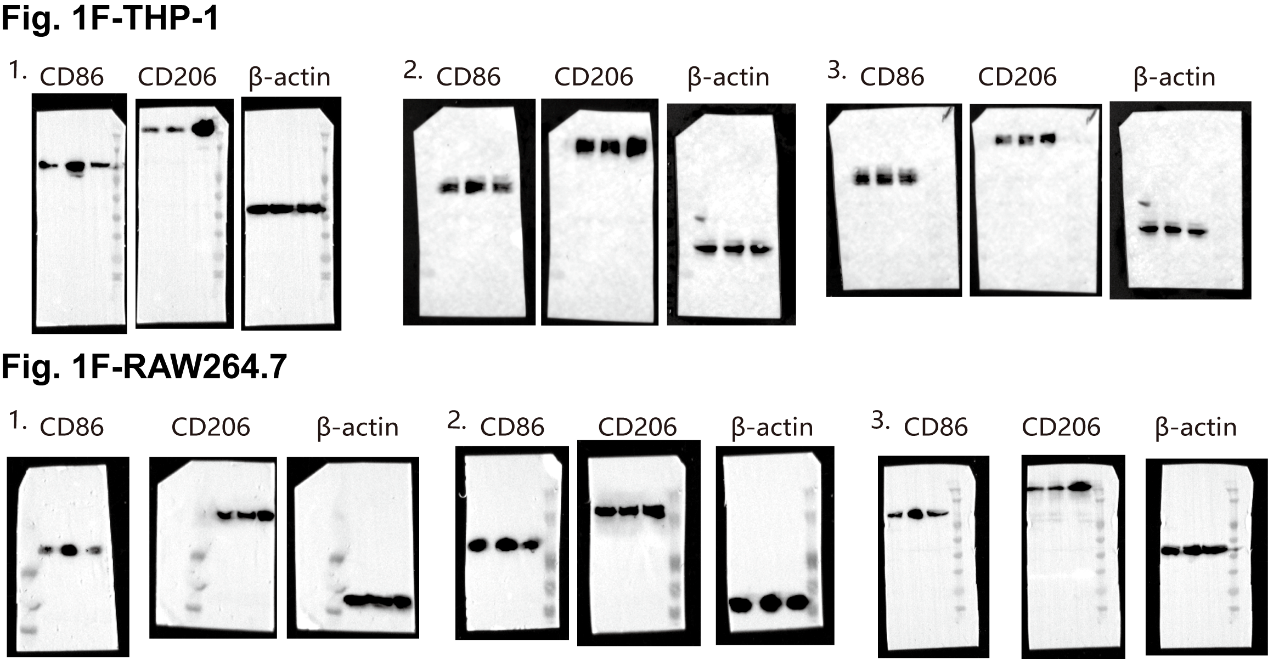


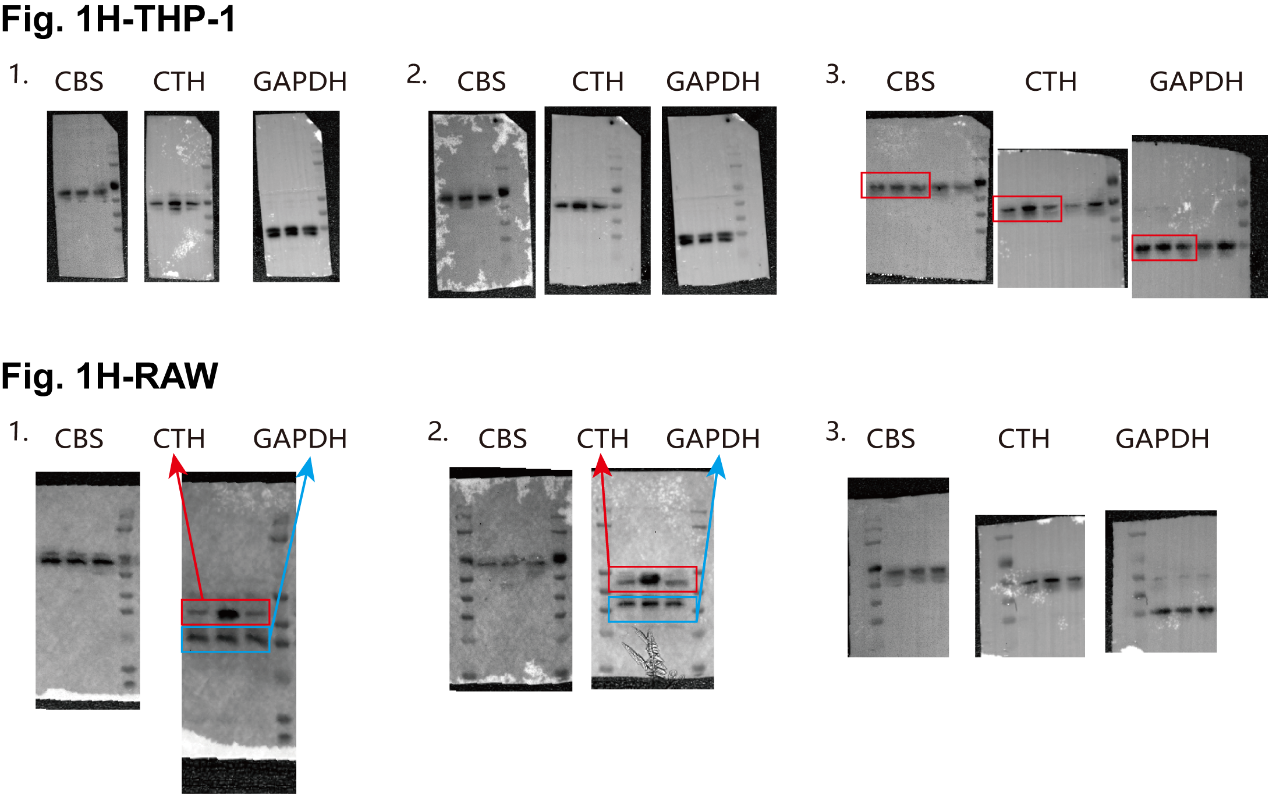

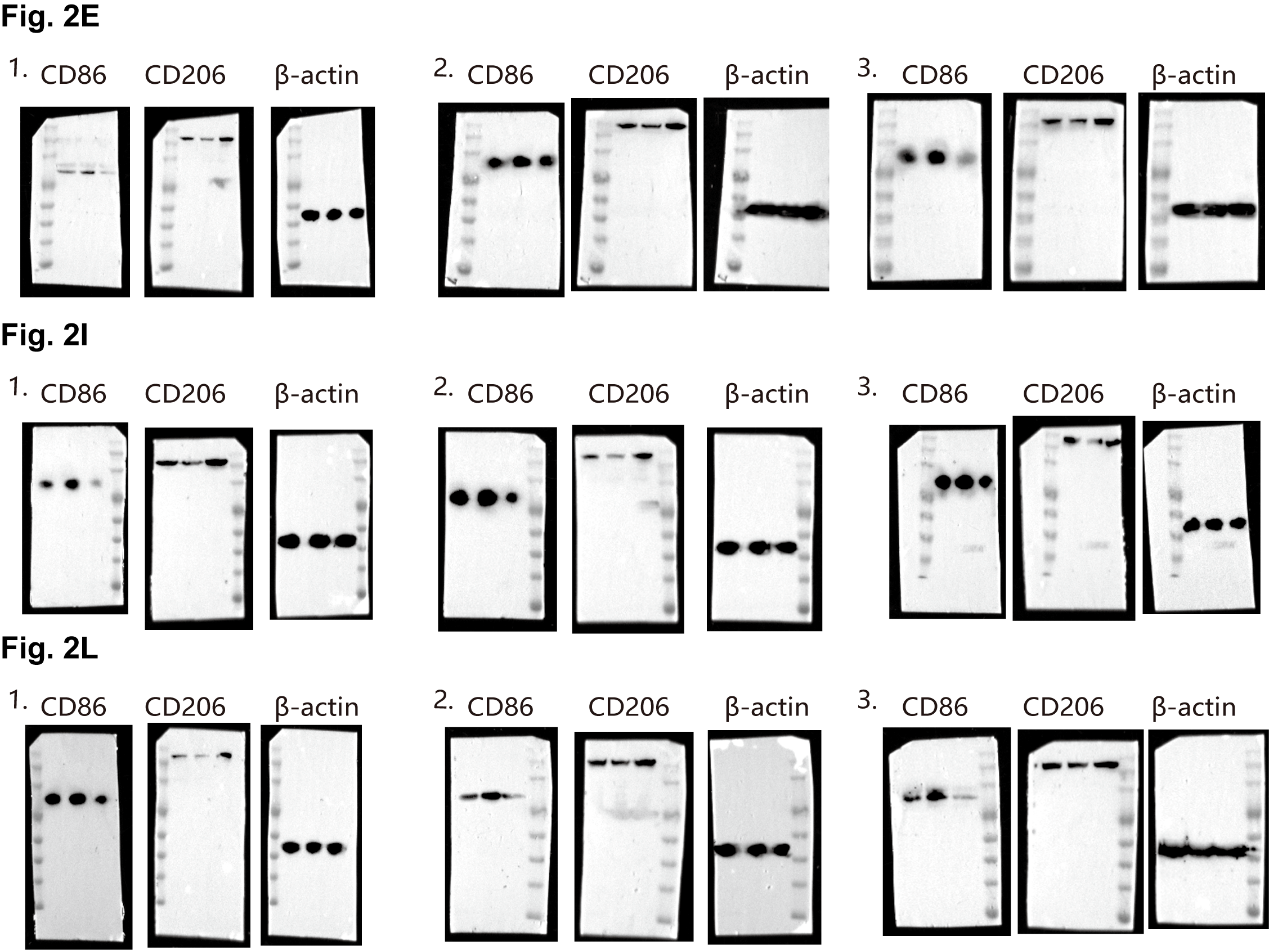

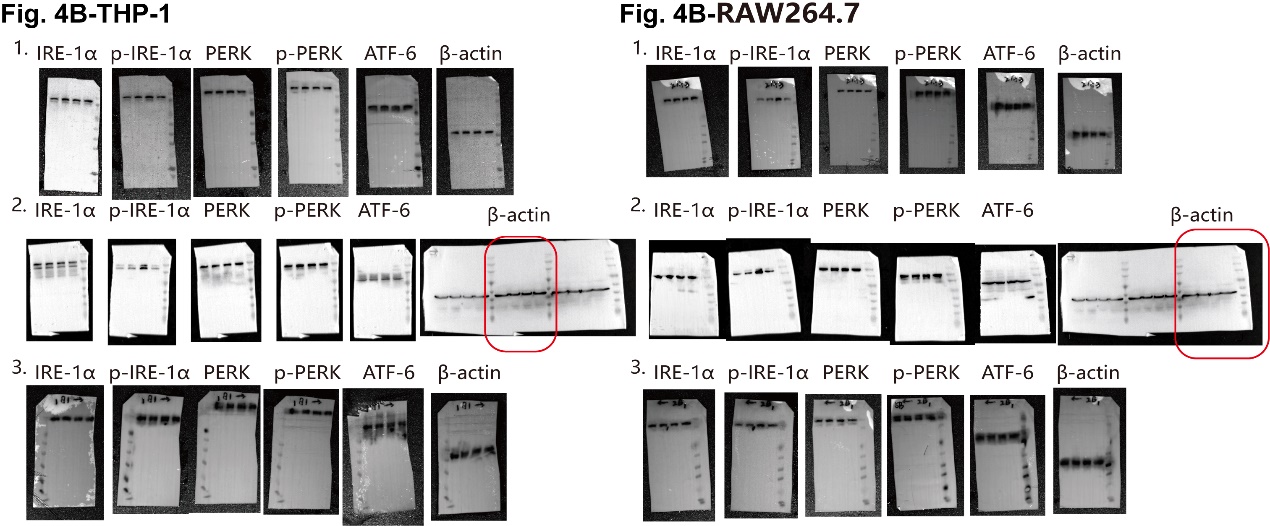

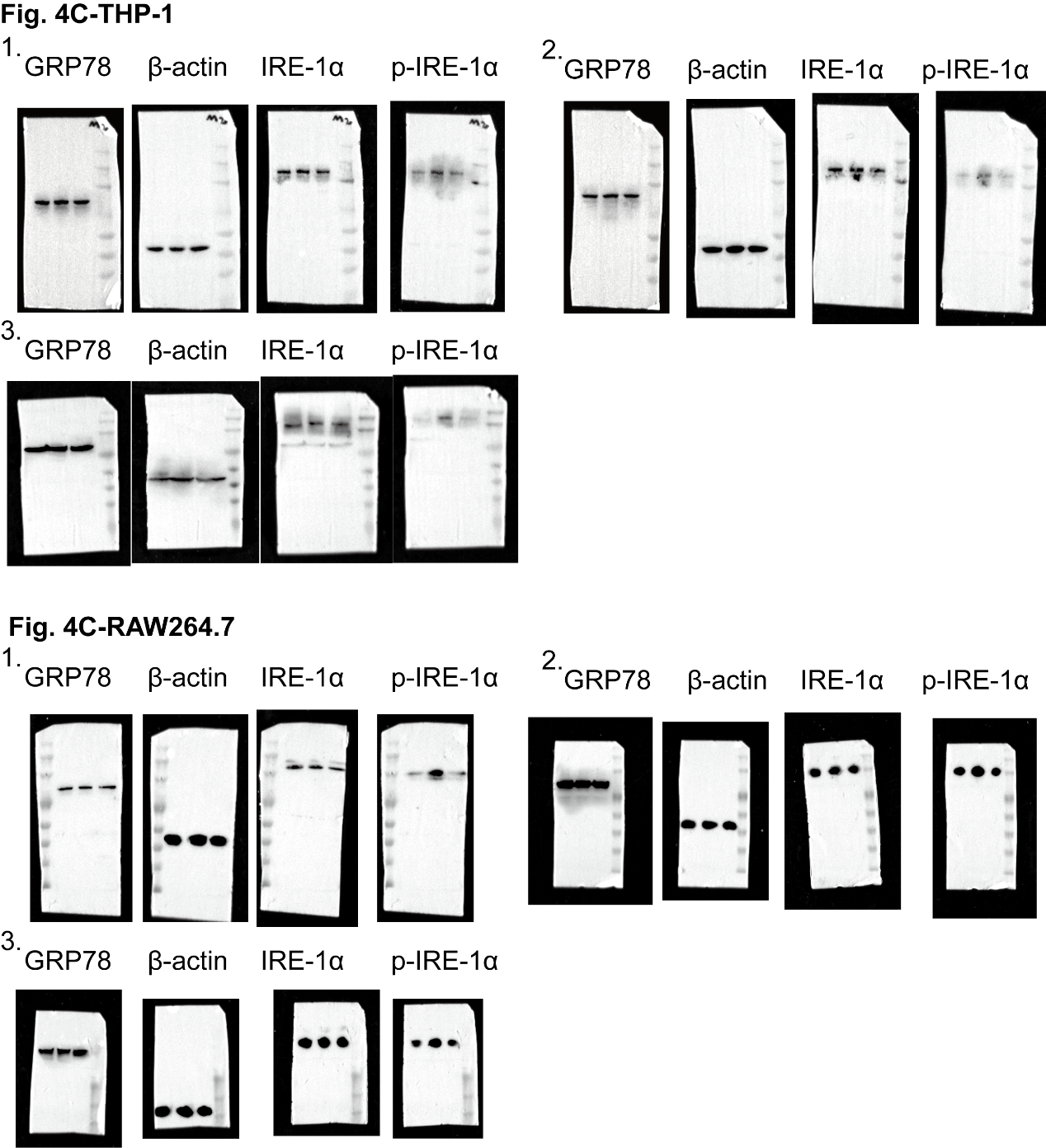

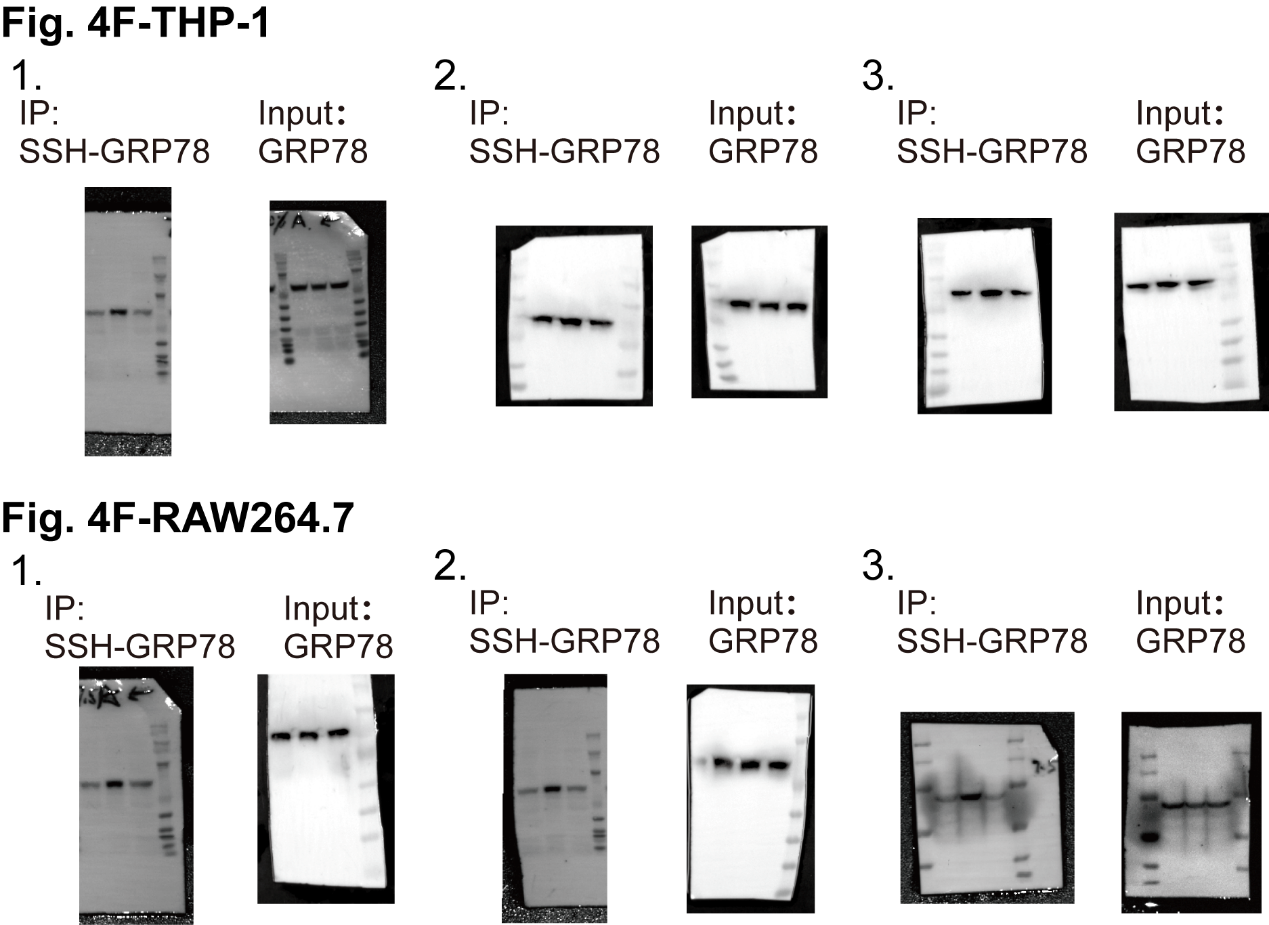

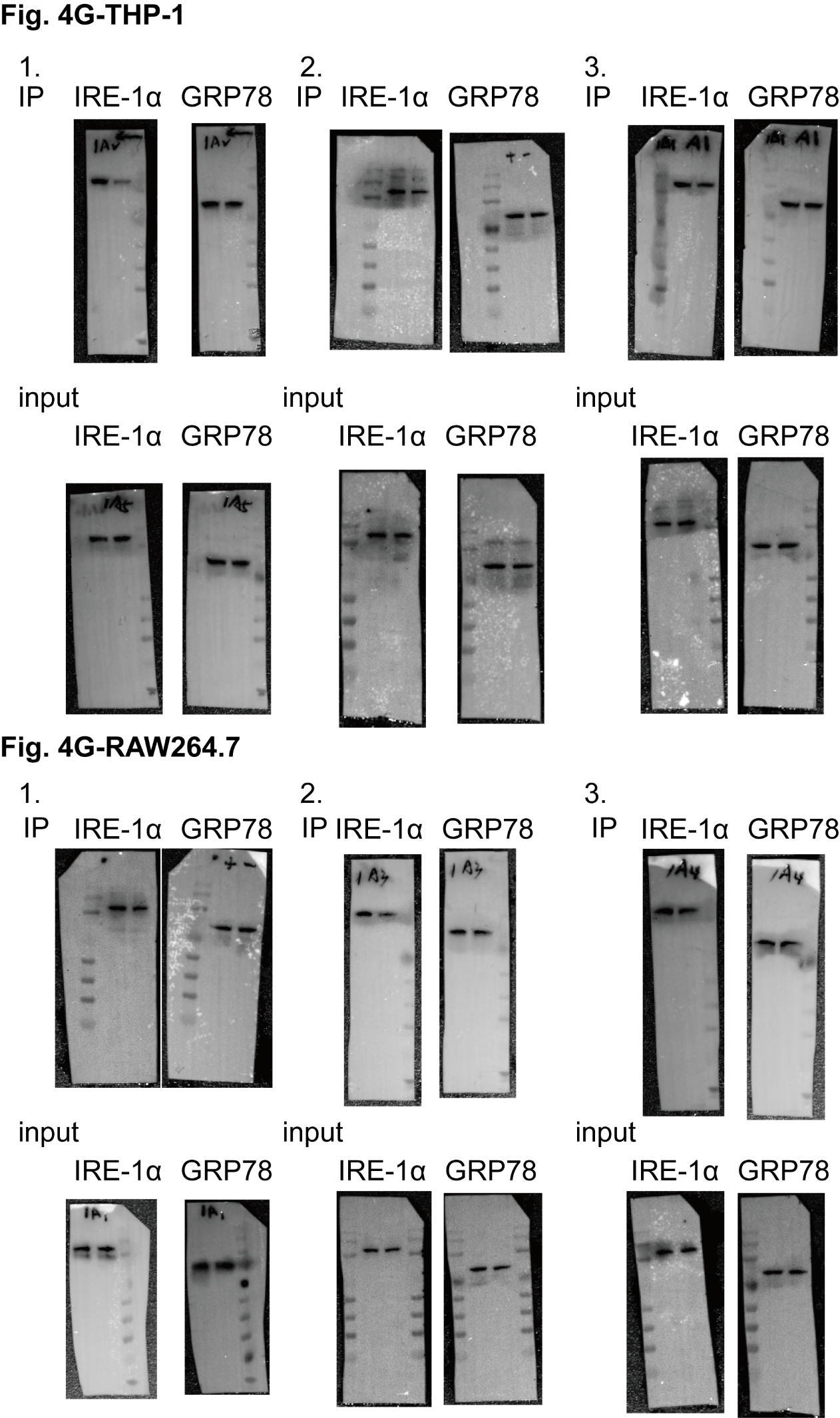

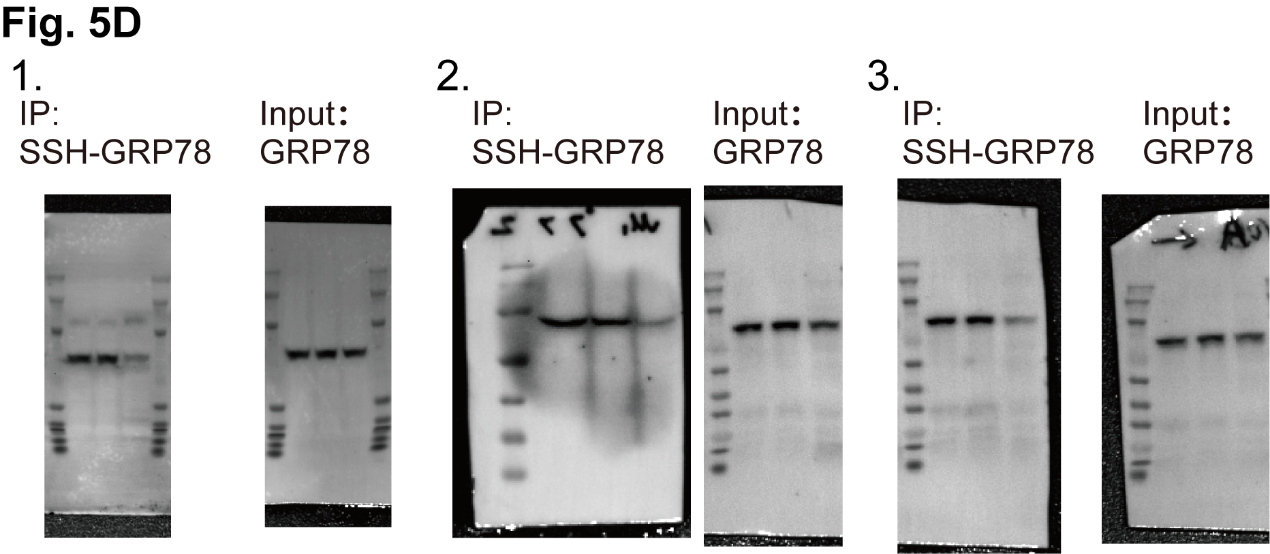

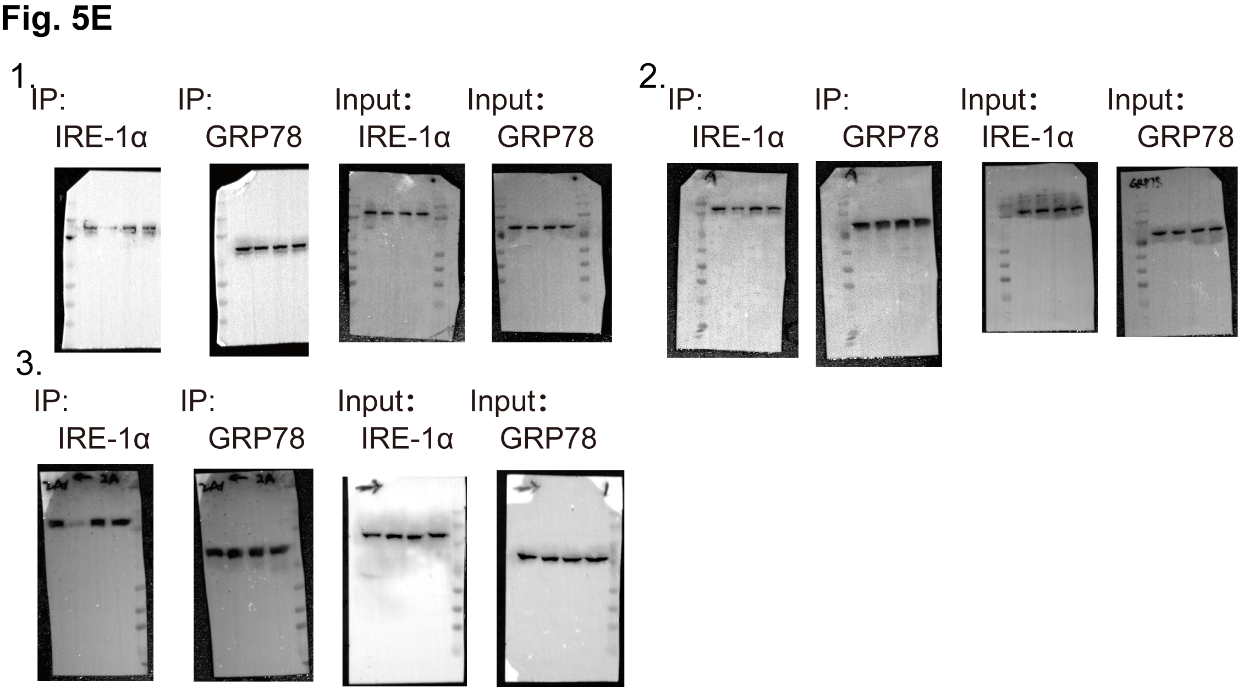

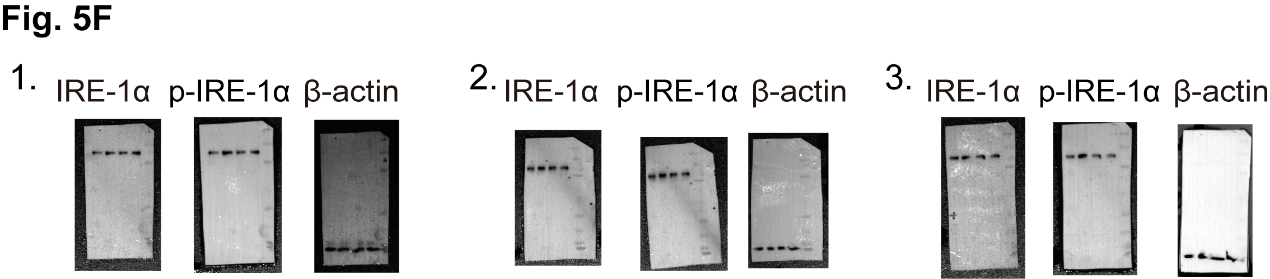

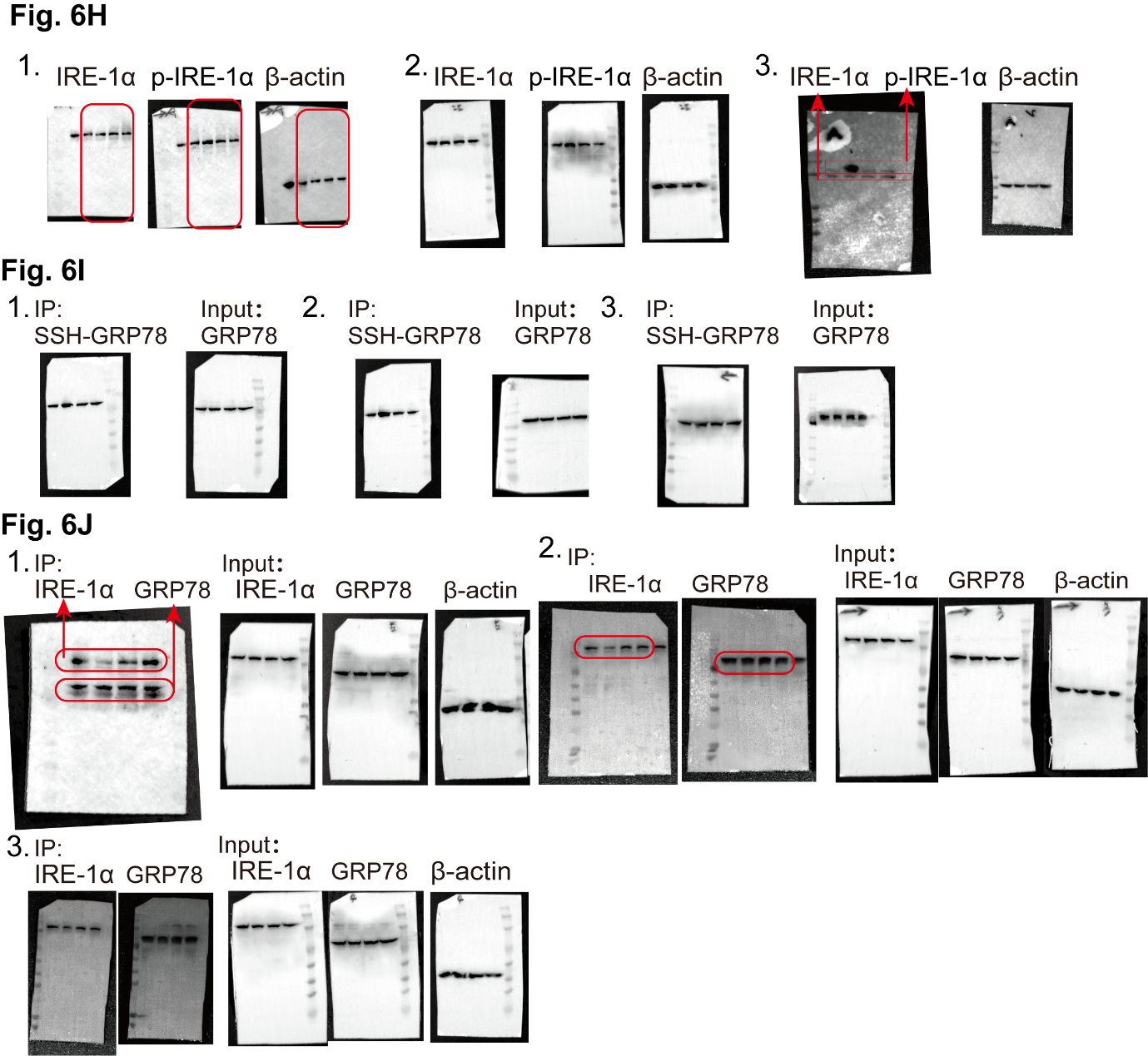

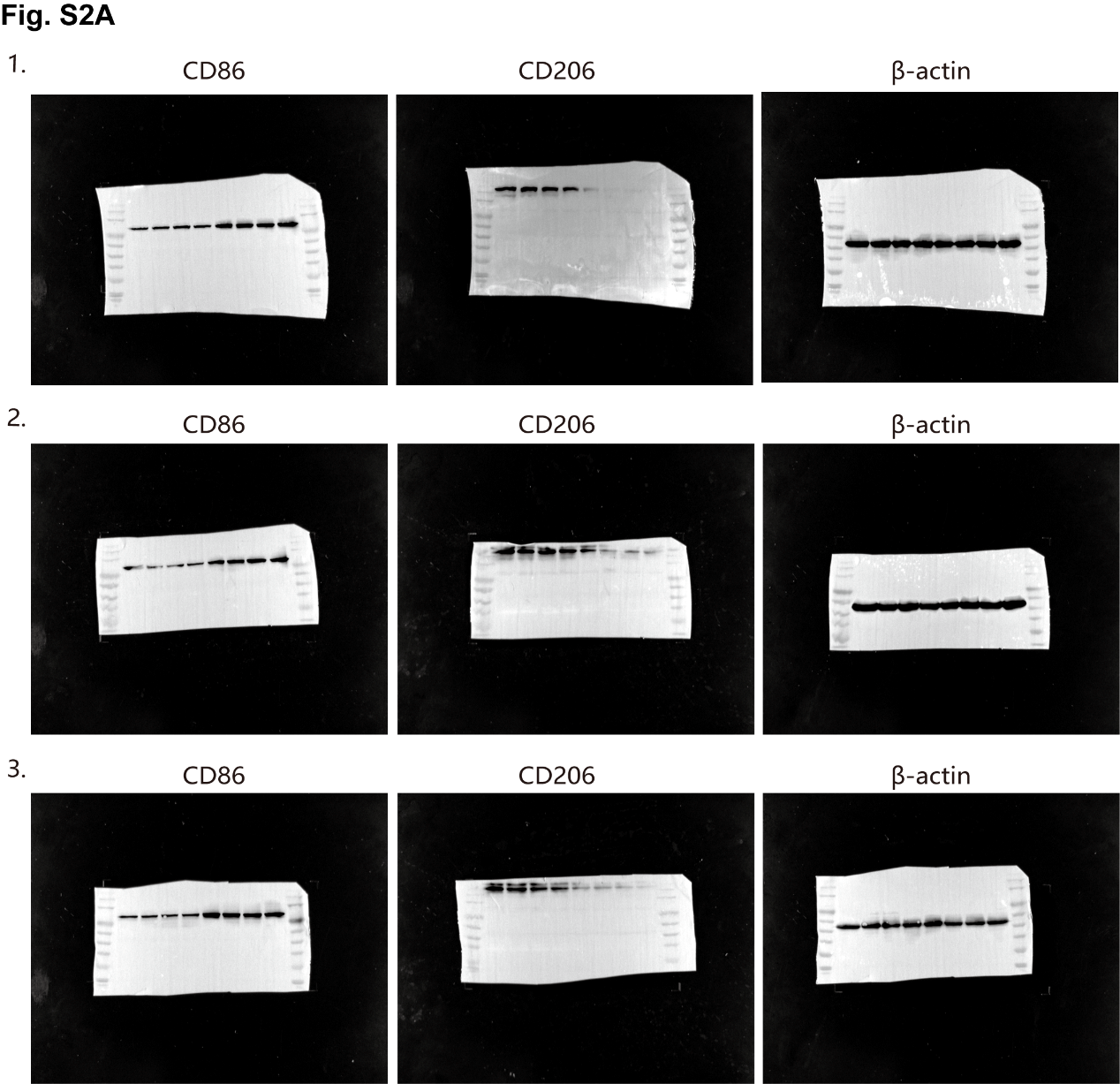

Supplement: Supplementary file 1 — Supporting Information [file ADVS-12-2413607-s001.docx]
